# Definitions of low cardiac output syndrome after cardiac surgery and their effect on the incidence of intraoperative LCOS: A literature review and cohort study

**DOI:** 10.3389/fcvm.2022.926957

**Published:** 2022-09-29

**Authors:** Anna Schoonen, Wilton A. van Klei, Leo van Wolfswinkel, Kim van Loon

**Affiliations:** ^1^Department of Anesthesiology, University Medical Center Utrecht, Utrecht, Netherlands; ^2^Department of Anesthesia and Pain Management Toronto General Hospital, University Health Network, Toronto, ON, Canada; ^3^Department of Anesthesiology and Pain Medicine, Temerty Faculty of Medicine, University of Toronto, Toronto, ON, Canada; ^4^Toronto General Hospital Research Institute, Toronto General Hospital, Toronto, ON, Canada

**Keywords:** low cardiac output syndrome, complication, definitions, incidence, cardiac surgery, LCOS

## Abstract

**Objectives:**

Low cardiac output syndrome (LCOS) is a serious complication after cardiac surgery. Despite scientific interest in LCOS, there is no uniform definition used in current research and clinicians cannot properly compare different study findings. We aimed to collect the LCOS definitions used in literature and subsequently applied the definitions obtained to existing data to estimate their effect on the intraoperative LCOS incidences in adults, children and infants.

**Design:**

This is a literature review, followed by a retrospective cohort study.

**Setting:**

This is a single-institutional study from a university hospital in the Netherlands.

**Participants:**

Patients from all ages undergoing cardiac surgery with cardiopulmonary bypass between June 2011 and August 2018.

**Interventions:**

We obtained different definitions of LCOS used in the literature and applied these to data obtained from an anesthesia information management system to estimate intraoperative incidences of LCOS. We compared intraoperative incidences of LCOS in different populations based on age (infants, children and adults).

**Measurements and main results:**

The literature search identified 262 LCOS definitions, that were applied to intraoperative data from 7,366 patients. Using the 10 most frequently published LCOS definitions, the obtained incidence estimates ranged from 0.4 to 82% in infants, from 0.6 to 56% in children and from 1.5 to 91% in adults.

**Conclusion:**

There is an important variety in definitions used to describe LCOS. When applied to data obtained from clinical care, these different definitions resulted in large distribution of intraoperative LCOS incidence rates. We therefore advocate for standardization of the LCOS definition to improve clinical understanding and enable adequate comparison of outcomes and treatment effects both in daily care and in research.

## Introduction

Low cardiac output syndrome (LCOS) is a frequently occurring complication after cardiac surgery. LCOS is characterized by an inadequate cardiac pump function resulting in reduced oxygen delivery and tissue hypoxia, in both adults and children (1). Clinicians may refer to LCOS for a symptomatic state that ranges from mild myocardial stunning to severe cardiogenic shock with the need for mechanical ventricular assistance. The reported incidence of LCOS varies from 2 to 27% in the adult population (2–8). In the pediatric population reported incidences are between 17 and 67% (9–11). Most studies describe the occurrence of LCOS, considering the associated morbidity (renal and pulmonary failure, stroke, myocardial infarction, sepsis, and a prolonged length of stay), mortality (up to 38%) and, therefore, increased healthcare costs (2–4, 6, 8, 12).

In order to properly address the features of LCOS that make it a potentially serious complication and to reduce its occurrence and seriousness, it is of crucial importance to study the syndrome. However, despite the obvious interest in LCOS from a clinical perspective, researchers do not use uniform criteria based on specific thresholds to describe the syndrome (definition) (13, 14). Several therapies have been evaluated for their effect on LCOS and compared in meta-analyses, however LCOS definitions differ among studies varying from the temporary use of a single vasoactive agent to counteract “stunning” to the requirement of mechanical support (15, 16). The comparison of study findings without the standardization of the LCOS definition including the use of uniform criteria (predefined thresholds) therefore is hampered.

We hypothesized that the variety in operational definitions of LCOS at least partly explains the wide range in reported incidences of LCOS. The primary aim of this study was to evaluate the variety of LCOS definitions described in literature among adult and pediatric cardiac surgery populations and subsequently to examine to what extent these different definitions affect the incidence of intraoperative LCOS.

## Methods

### Design and conduct of the study

In this study, we combined a literature review approach with a retrospective cohort study. A study protocol was not published nor registered. The literature review was used to extract LCOS definitions. Subsequently, we applied the definitions found to a retrospective intraoperative cohort, to study the effects of the different definitions on the estimated incidence of LCOS. The cohort included cardiac surgery with cardiopulmonary bypass patients from all ages, i.e., both children and adults.

### Review of the literature

The following literature search was performed in the PubMed database (17) on August 24th, 2020:

(((((Low cardiac output syndrome[Title/Abstract]) OR LCOS[Title/Abstract])) AND (((((((((Surger^*^) OR Operation^*^) OR Surgical procedure^*^) OR operative surgical procedure^*^) OR operative procedure^*^)) AND ((heart) OR cardiac))) OR ((((“Heart”[Mesh]) AND “Surgical Procedures, Operative”[Mesh])) OR “Cardiac Surgical Procedures”[Mesh])))).

We excluded articles when the full text was not available, those written in non-English language, those including a non-human study population and duplicate papers. We also excluded articles without a definition of LCOS (for example where LCOS was not a main outcome), systematic reviews, case reports, editorials, author's opinions and letters to the editor. We did not exclude articles based on publication year. The remaining articles were reviewed, the definitions of LCOS were extracted and categorized. We classified the articles on the following items: study population (adult/pediatric/both/questionnaires completed by pediatric Intensive Care Unit (PICU) professionals), reproducibility and scope. Definitions included “inotropes,” “cardiovascular mechanical support,” “acidosis,” “cardiac pump function,” “blood pressure,” “clinical signs of hypoperfusion,” “saturation,” “pulmonary capillary wedge pressure,” “renal replacement therapy,” “hemodynamic instable,” “cardiac arrest,” “death,” and “others.” Definitions were listed as reproducible, when they had cut-off values in their definition and when they did not use vague terms without further explanation, such as “a situation, in which circulation and organ perfusion is barely maintained” (18). Detailed information about selection, data extraction, and scoring is provided in Supplementary material 1. Screening, selection and data extraction was done by author AS and in case of uncertainty discussed with KL.

### Retrospective cohort study

We listed the 10 most frequently published LCOS definitions and used these to determine the intraoperative incidence of LCOS according to these definitions. The study data were collected from the University Medical Center Utrecht (UMCU; The Netherlands) and the Wilhelmina Children's hospital, which is part of the UMCU. The UMCU is a tertiary referral hospital for pediatric and adult cardiac surgery. The study population included patients of all ages who underwent cardiac surgery with cardiopulmonary bypass (CPB) between June 2011 and August 2018. Our center performs the full range (Basic Aristotle score 1.5–15) of congenital cardiac surgery, including neonatal Norwood procedures, with an average Basic Aristotle score of 7.06 (SD 2.97). We did not use postoperative intensive care data as the AIMS and intensive care databases were not connected, nor similarly constructed. The Medical Ethics Review committee reviewed the study protocol and waived the need for patient consent (WAG/rgj/18/022047).

We collected the following variables: age, gender, weight, height, type of surgery, surgical urgency, duration of CPB, vital parameters, laboratory tests, inotropic drugs administration and in-hospital mortality. Patient characteristics, laboratory tests and in-hospital mortality were obtained from the Electronic Medical Record system (HIX 5.2, ChipSoft, Amsterdam, The Netherlands). Intraoperative data were obtained from the anesthesia information management system (AIMS) database (Anstat, Carepoint, Ede, The Netherlands).The first postoperative laboratory test results of lactate, arterial pH, arterial oxygen saturation, mixed venous oxygen saturation and central venous oxygen saturation were used. Variables extracted from the AIMS database were stored as median per minute values during the intraoperative period. These variables included intraoperative vital signs, anesthesia ventilator data and data on inotropic use. For the vital parameters and inotropic drugs, we used the mean, timeframes, or limits defined otherwise of all variables stored in the AIMS after the patients were weaned from the CPB. We used the 50th percentile of the national growth charts to estimate the height of children, because the documentation of this variable was unreliable (19). We considered other missing data to occur under the “missing not at random” condition as cardiac vascular monitoring and treatment is initiated based on clinical indication. Therefore, no further missing data assumptions were made.

The primary outcome was the difference in incidence of LCOS. We applied the 10 most frequently reported LCOS definitions to the data obtained to determine the incidences. To calculate LCOS incidences, we used the total population as the denominator (i.e., the patients with and without missing data) to prevent biased estimates due to selective missingness of data. When LCOS definitions were built with “*OR*” condition statements (e.g., “the use of inotropes *OR* mechanical support”), patient data were considered missing only if all parts of the statement could not be filled in (e.g., in this case, patient data were only considered missing if there was no data available on the use of inotropes *AND* no data on mechanical support).

As secondary outcome, we compared incidences between adults (≥18 years), children (>6 months and <18 years) and infants (≤6 months). We chose 6 months as cut-off point between infants and children, because maturation of the human heart is completed at 6 months of age, anatomically (remodeling of pulmonary blood flow and closure of the foramen ovale, ductus venosus and ductus arteriosus), histologically (growth of mitochondria numbers, myofibrils numbers and sarcomere volume and development the sarcoplasmic reticulum) and physiologically (increasing the coronary oxygen supply and preload due to a decreased heartrate) (20).

### Statistical analysis

The statistical analysis was performed with R-studio software, version 1.1.456 (21). Continuous variables are presented as means ± standard deviations (SD) or when skewness or kurtosis was observed, as medians with interquartile ranges (IQR). Categorical variables are presented as proportions.

## Results

### Literature search

**Figure 1:**
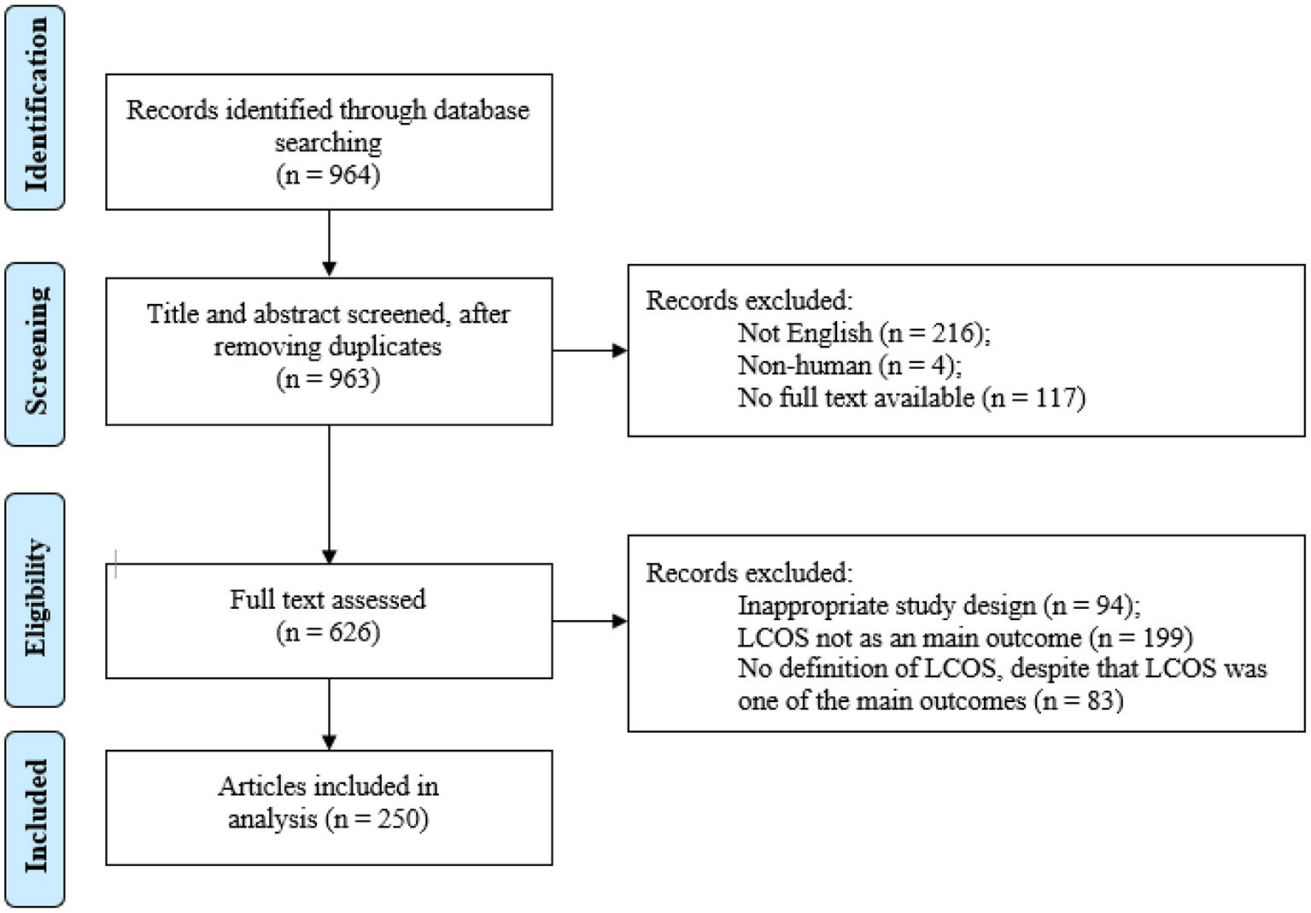
Literature search. LCOS, low cardiac output syndrome.

The literature search identified 964 records that were handled as presented in Figure 1. Ultimately, 250 articles were included (Supplementary material 2). In five of these articles multiple definitions were used (5 (15), 5 (9), 2 (22), 3 (23) and 2 (24), respectively), initially resulting in a total of 262 definitions.

Of the 262 included definitions, 177 (68%) focused on adult surgery, 80 (31%) focused on pediatric surgery and 5 (2%) used information gained by questionnaires completed by pediatric ICU professionals as study population. Of the 262 definitions, 175 definitions (67%) were reproducible, i.e., definitions were clearly described and used cut-off values, and 87 definitions (33%) were not. Twelve items were repeatedly used within the LCOS definitions, namely: the use of inotropes; mechanical support; metabolic acidosis; cardiac pump function; blood pressure; clinical signs of hypoperfusion; saturation; pulmonary capillary wedge pressure (PCWP); renal replacement therapy; clinical judgement; cardiac arrest and death (Table 1). The definition of LCOS in studies among adults more often included need for mechanical support and cardiac pump function, while definitions used in pediatric studies more often included metabolic acidosis and clinical signs of hypoperfusion (Table 1).

**Table 1:** Variables used in low cardiac output syndrome definitions.

	**Definitions in a study with an adult study population, *n* (%)**	**Definitions in a study with a pediatric study population, *n* (%)**	**Definitions in a study where PICU workers were interviewed, *n* (%)**
**No. of definitions**	177 (100)	80 (100)	5 (100)
**Reproducible**	135 (76)	39 (49)	1 (20)
**Inotropes**	126 (71)	49 (61)	3 (60)
Duration of the use of inotropes	69 (39)	7 (8.8)	0 (0.0)
Type of inotropes	45 (25)	7 (8.8)	0 (0.0)
VIS	2 (1.1)	13 (16)	0 (0.0)
Number of used inotropes	13 (7.3)	19 (24)	3 (60)
**Mechanical support**	94 (53)	16 (20)	0
ECMO	11 (6.2)	6 (7.5)	0
IABP	80 (45)	2 (2.5)	0
VAD	5 (2.8)	2 (2.5)	0
Not specified	14 (7.9)	8 (10)	0
**Metabolic acidosis**	11 (6.2)	51 (64)	3 (60)
High lactate	7 (4.0)	38 (48)	1 (20)
High base difference	0	13 (16)	0
Low pH	0	4 (5.0)	0
Low bicarbonate	0	3 (3.8)	0
**Cardiac pump function**	115 (65)	16 (20)	0
Low CI	114 (64)	9 (11)	0
Low left ventricle ejection fraction	1 (0.6)	6 (7.5	0
**Blood pressure**	70 (40)	20 (25)	1 (20)
Low systolic blood pressure	58 (33)	7 (8.8)	0
Low mean arterial pressure	5 (2.8)	4 (5.0)	0
Low central venous pressure	4 (2.3)	2 (2.5)	0
High systemic vascular resistance	2 (1.1)	1 (1.3)	0
**Clinical signs of hypoperfusion**	32 (18)	47 (59)	5 (100)
Oliguria	20 (11)	43 (54)	2 (40)
Tachycardia	4 (2.3)	33 (41)	3 (60)
Cold extremities	16 (9.0)	35 (44)	2 (40)
Altered mental state	10 (5.6)	2 (2.5)	0
Clammy skin	4 (2.3)	0	0
Others	0 (0.0)	3 (3.8)	0
**Decreased oxygen saturation**	11 (6.2)	28 (35)	1 (20)
High difference between arterial and venous saturation	0	22 (28)	1 (20)
Low arterial oxygen pressure	3 (1.7)	0	0
Low venous oxygen saturation	9 (5.1)	10 (13)	0
**PCWP**	16 (9.0)	0	0
**Renal replacement therapy**	2 (1.1)	1 (1.3)	0
**Clinical judgement**	9 (5.1)	1 (1.3)	0
**Cardiac arrest**	0	22 (28)	1 (20)
**Death**	2 (1.1)	6 (7.5)	0
**Other**	9 (5.1)	9 (11)	0

PICU, pediatric intensive care unit; VIS, vasoactive-inotropic score; ECMO, extracorporeal membrane oxygenation; IABP, intra-aortic balloon pump; VAD, ventricle assistant device; CI, cardiac index; PCWP, pulmonary capillary wedge pressure.

The need of inotropes was used in the definition in 71 and 61% of the articles including adult and pediatric cardiac surgery patients, respectively. There were four different ways in which inotropes were used in definitions, namely: (1) the duration of inotrope administration; (2) the specific inotropic drug used (epinephrine, norepinephrine, dopamine, dobutamine and milrinone); (3) the Vasoactive-Inotropic Score (VIS), a formula that quantifies the amount of cardiovascular support (25, 26); and (4) the number of used inotropes (Table 1). In studies involving the adult population, the cut-off value for the duration of inotrope administration in LCOS definitions was shorter (median 2.0 h) than for the studies involving the pediatric population (median 24.0 h). Compared to the pediatric population, in studies including adults, the inotropic drug was more often specified (25 vs. 9%) and VIS and the number of inotropes used were less likely used in the definition (1 vs 16%, and 7 vs 24%, respectively).

### Cohort study

Between June 2011 and August 2018, 7,366 patients underwent cardiac surgery with cardiopulmonary bypass. The cohort included 5,934 (80.6%) adults, 690 (9.4%) children and 742 (10.1%) infants (Table 2). In all groups, there were more males than females. In adults, coronary artery bypass grafting (CABG) was the most frequently performed procedure. Repairs for atrial septal defects and ventricle septal defects were performed most frequently in the pediatric and infant group, respectively. Adults and infants had a higher in-hospital mortality rate than children: 3.1, 3.0, and 0.7%, respectively.

**Table 2:** Patient demographics and baseline characteristics.

	**Adults (*N* = 5,934)**	**Children (*N* = 690)**	**Infants (*N* = 742)**
**Male sex, *n* (%)**	4,136 (70)	368 (53)	432 (58)
**Age, median (IQR)**	66 (56–73) years	3.0 (1.0–9.0) years	2.0 (0.0–4.0) months
**Body surface area, median (IQR)**	1.96 (1.8–2.1)	0.8 (0.5–1.1)	0.3 (0.2–0.3)
**Urgency of surgery**, ***n*** **(%)**
Elective	5,383 (91)	677 (98)	710 (96)
Emergency	551 (9.3)	13 (1.9)	32 (4.3)
Reoperation, *n* (%)^*^	255 (4.3)	90 (13)	121 (16)
Type of surgery, *n* (%) ^**^	CABG 2,462 (41.5)	ASD surgery 135 (19.6)	Combined congenital surgery 160 (21.6)
	AV surgery 584 (9.8)	VSD surgery 87 (12.6)	VSD surgery 118 (15.9)
	Combined CABG and AV surgery 475 (8.0)	Combined congenital surgery 82 (11.9)	Tetralogy of Fallot 91 (12.3)
	MV surgery 304 (5.1)	PV surgery 74 (10.7)	Arterial switch operation 62 (8.4)
	Thoracic aortic surgery 201 (3.4)	Repair of anomalous pulmonary venous connection 71 (10.3)	Repair of anomalous pulmonary venous connection 54 (7.3)
**Duration of operation (min), median (IQR)**	282 (239–351)	247 (197–310)	282 (230–337)
**Length of CPB (min), median (IQR)**	107 (78–158)	68 (48–114)	113 (72–147)
**Need of MCS, *n* (%)**	316 (5.3)	4 (0.6)	3 (0.4)
**30-day mortality, *n* (%)**	182 (3.1)	5 (0.7)	22 (3.0)

* Reoperation was defined as any patient, who had more than one cardiac surgery in our institution between June 2011 and August 2018.

** In this table we only show the five most frequently performed procedures.

IQR, interquartile range; CABG, coronary artery bypass grafting; AV, aortic valve; MV, mitral valve; ASD, atrial septum defect; VSD, ventricle septum defect; PV, pulmonary valve; min, minutes; CPB, cardiopulmonary bypass; MCS, mechanical circulatory support.

### Incidences of low cardiac output syndrome

We used the complete cohort (5,934 adults, 690 children, 742 infants) as denominator within the LCOS incidence calculation for the 10 most frequently published definitions. Tables 3, 4 show the number of patients available to count the LCOS cases in the numerator. We were unable to calculate the incidences for all definitions due to missing not at random (MNAR) data. As an example, children and infants did not receive invasive cardiac output monitoring (such devices are not intended nor validated for pediatric use) and no extracorporeal circulation devices like ventricular assist or intra-aortic balloon counterpulsation (IABP) devices.

**Table 3:** Top 10 most published definitions of low cardiac output syndrome.

	Definitions^*^	**Appearance of definition in articles *n* (%)**	**Articles concerning adults [A], children [C] or both [AC]**
1	Cardiac index < 2.0 L/min/M^2^	16 (5.9) (27–42)	[AC]
2	Duration of inotropic use for >30 min with specified types of inotropes to maintain a systolic blood pressure >90 mmHg^*^; or the use of IABP; or a cardiac index < 2.2 L/min/M^2^	13 (4.8) (2–4, 8, 43–51)	[A]
3	Duration of inotropic use for >30 min to maintain a systolic blood pressure 90 mmHg^*^; or the use of IABP; or cardiac index < 2.2 L/min/M^2^	9 (3.3) (52–60)	[A]
4	The use of more than one inotropic drug and a lactate >2.0 mmol/L	6 (2.2) (9, 15)	[C]
5	Duration of inotropic support for >24 h; or a cardiac index < 2.0 L/min/M^2**^	6 (2.2) (6, 61–65)	[A]
	Metabolic acidosis (a lactate >2.0 or base difference >4.0) with clinical signs of hypoperfusion (tachycardia >90/min^*^ or oliguria 0.5 ml/kg/h); or an arterial-venous saturation difference >30% with clinical signs of hypoperfusion (tachycardia >90/min^*^ or oliguria 0.5 ml/kg/h); or a cardiac arrest	6 (2.2) (66–71)	[C]
7	Cardiac index < 2.0 L/min/M^2^ and an increased PCWP	6 (2.2) (12, 72–75)	[A]
8	Cardiac index < 2.0 L/min/M^2^ despite the use of inotropes (not further specified); or the use of IABP	5 (1.8) (76–80)	[A]
9	The use of inotropes (not further specified); or mechanical support (ECMO, IABP or VAD)	5 (1.8) (81–85)	[A]
10	Mechanical support (ECMO, IABP or VAD)	5 (1.8) (86–90)	[A]

* If articles used different cut-off values, e.g., cardiac index cut-off values ranched from 1.75 till 3.0, we present the median cut-off value.

IABP, intra-aortic balloon pump; PCWP, pulmonary capillary wedge pressure; ECMO, extracorporeal membrane oxygenation; VAD, ventricle assistant device.

**Table 4:** Top 10 most published definitions of Low Cardiac Output Syndrome (LCOS) and corresponding incidence rates in an intraoperative cohort with adults, children and infants.

**Top 10 LCOS definitions (see Table 3 for definitions)**	**Adults (*****N*** = **5,934)**		**Children (*****N*** = **690)**		**Infants (*****N*** = **742)**	
	**No. of patients without missing data**	**Incidence LCOS in %**	**No. of patients without missing data**	**Incidence LCOS in %**	**No. of patients without missing data**	**Incidence LCOS in %**
1	400	1.5	0	–	0	–
2^*^	5,667	8.3	516	23.8	534	30.1
3^*^	5,667	31.5	516	27.4	534	32.3
4	2,049	12.3	688	5.1	739	16.3
5^**^	–	–	–	–	–	–
6^*^	3,365	13.7	542	6.4	571	14.4
7	0	–	0	–	0	–
8	414	1.8	0	–	0	–
9	5,934	91.0	690	55.7	742	82.2
10	5,934	4.5	690	0.6	742	0.4

* For systolic hypotension and tachycardia we used for children and infants the p-values (91, 92). The p5 for systolic hypotension and p90 for tachycardia.

** Intensive care follow-up data was not available (e.g., maximum duration of inotropic support). Hence, we could not determine the incidence of LCOS using this definition.

LCOS, low cardiac output syndrome.

In all three groups (adults, children and infants), applying the definition “*The need of inotropic use* *OR* *mechanical support (IABP, VAD or ECMO)*” resulted in the highest LCOS incidence: 91.0% in adults, 55.7% in children and 82.2% in infants, respectively (Table 4). The definition “Cardiac index <2.0 L/min/M^2^” resulted in the lowest incidence in adults (1.5%). The definition “*The need for mechanical support”* resulted in the lowest incidences in children and infants, respectively 0.6 and 0.4%. Definitions without missing data were “*the need of inotropic use or mechanical support”* and “*the need of mechanical support.”*

## Discussion

This study summarized different criteria used for the definition of LCOS described in literature and subsequently estimated the incidence of LCOS immediately after surgery by applying these definitions to a large patient cohort. We found 171 different definitions and using the 10 most frequently reported ones resulted in an estimated incidence of intraoperative LCOS ranging from 1.5%−91% and 0.6%−56% in adults and in children, respectively. To the best of our knowledge, this is the first article to focus on the description and use of different LCOS definitions.

Low cardiac output syndrome, caused by an inadequate cardiac pump function, is a serious complication after cardiac surgery with high morbidity and mortality (2–4, 6, 8, 12). Far-reaching scientific interest in different kinds of interventions and their effect on LCOS have resulted in numerous publications on the subject. Many studies used the incidence of LCOS as a primary outcome. Although the criteria used to define LCOS were reported in most articles, these were frequently not reproducible (34%) and most articles did not explain why specific criteria were chosen. This resulted in the use of pluriform criteria to define the syndrome. In our study, we found a striking total of 171 different variations to define LCOS. Furthermore, we noticed that definitions used also greatly differed between the adult and pediatric populations. Our study demonstrated that the definition of LCOS is a very important explanatory determinant for the reported incidence of LCOS.

Currently, there is no uniform definition of LCOS, despite the presumed importance that clinicians use the same language and the generalizability of future scientific evaluations of new therapies. Therefore, we question whether LCOS should be used to describe an inadequate cardiac pump function after surgery without taking the necessary step toward uniformity. A uniform definition ensures that there will be fewer reasoning errors, misunderstandings, unnecessary controversies or problems in comparing scientific results. We argue that a good definition should be reproducible, generally valid among different populations and measurements should be as less invasive as possible (minimizing the risk of side effects). Abstract concepts, vague terminology and measurements without cut-off values result in unreproducible definitions with an immediate effect on the incidence rate of a certain outcome like LCOS. From our literature review, 34% of the definitions were not reproducible, due to lack of cut-off values or vague terminology. None reproducible criteria hamper generalizability of findings and re-evaluation of study results. Furthermore, definitions should preferably not rely on invasive monitoring technology that is only used in high risk populations, especially if there are good alternatives without those consequences. For example, the pulmonary capillary wedge pressure was frequently used in the definitions of LCOS (10% of the articles with an adult cardiac population). A Pulmonary Artery Catheter gives an inherent risk of mechanical, thrombotic, and infectious complications (93, 94) and is therefore only used in complex cardiac cases. These practical issues bias the incidence of LCOS as a significant part of the study population never adheres to the criteria because PCWP or CI were not routinely measured. Also, PCWP and CI were not measured in children because of technical impossibilities and the unavailability of validated monitoring equipment for pediatric use.

Another interesting finding of this study is the apparent difference between definitions used in adults and children. These differences elicit the question whether we are describing two different disease entities with maybe also a different biochemical origin. For LCOS in adults, studies used the items “mechanical support” and “cardiac pump function” more often, whereas in children, studies frequently used the items “metabolic acidosis” and “clinical signs of hypoperfusion.” Although it needs no explanation that pediatric patients differ from adult cardiac surgery patients, in both settings a valid definition for post-bypass inadequate cardiac pump function or LCOS is valuable and may contribute to the generalizability of scientific work.

Our study certainly has some limitations. First, our literature review was executed in PubMed and other databases were not searched for, and we thus might have missed relevant publications, and we did not publish nor register the study protocol. Second, this is retrospective review of a single-center experience and so this implicates that we could not track down the reasons behind inotropic use, mechanical support and others. We also had to assume the association between the therapeutic interventions and vital parameters measured which might limit generalizability. Third, we did not have any information about observations like cold extremities or altered state of mind, as we used intraoperative data and these were uncommonly observed and documented in the AIMS. As a result, we expressed the clinical signs of hypoperfusion solely with tachycardia and oliguria. Fourth, we had missing data for some of the items in the LCOS definitions that were time dependent. We only used intra-operative data, collected from our AIMS and no postoperative follow-up data, while the peak incidence of LCOS is expected 6–12 h after cardiac surgery. For that reason also, we were unable to calculate the incidence of LCOS in one of the 10 most frequently published definitions, that used inotropic support for 24 h after cardiac surgery as criterion. These limitions may cause under- and/or overestimations of the incidence of LCOS. However, because our primary outcome was the difference in incidence of LCOS using different definitions, we still report these numbers to show the effect different definitions have on the incidence. Finally, most definitions are also applicable during postoperative admission. We did not collect data about decreased cardiac indices or decreased systolic blood pressures at the ICU, which may be considered as a major limitation of our study. However, this study should not be used as a reference for LCOS occurrence after cardiac surgery, because we mainly aimed to illustrate the effect of the absence of a uniform LCOS definition in daily practice. Our study results could additionally serve to compare different age groups.

We suggest that consensus should be reached about a reproducible and practical LCOS definition within and between the international scientific societies. Prospective research that evaluates this universal LCOS definition would help to understand the features and occurrence of the syndrome in adults, children and infants. Furthermore, studies (95) in which LCOS is precursor for poor outcome, would enable the use of LCOS as a useful surrogate endpoint.

## Conclusion

This study collected different definitions of LCOS and evaluated how they influenced estimations of the intraoperative incidence of LCOS in adults, children and infants. From the 171 different kind of definitions found, we used the 10 most frequently published and applied these to a large sized cohort including patients from all ages. We calculated LCOS incidence estimates ranging form 0.4 to 91%. We would like to advocate for standardization of the LCOS definition to improve clinical understanding and enable adequate comparison of outcomes and treatment effects both in daily care and in research.

## Data availability statement

The original contributions presented in the study are included in the article/Supplementary material, further inquiries can be directed to the corresponding author.

## Author contributions

AS and KvL contributed to conception and design of the study, performed the statistical analysis and wrote the first draft of the manuscript. LvW designed and completed the database for the retrospective cohort study. WvK contributed with a thorough review and rewriting of the manuscript draft. All authors contributed to manuscript revision, read and approved the submitted version.

## Funding

WK is supported by the R. Fraser Elliott Chair in Cardiac Anesthesia (Toronto, ON, Canada).

## Conflict of interest

The authors declare that the research was conducted in the absence of any commercial or financial relationships that could be construed as a potential conflict of interest.

## Publisher's note

All claims expressed in this article are solely those of the authors and do not necessarily represent those of their affiliated organizations, or those of the publisher, the editors and the reviewers. Any product that may be evaluated in this article, or claim that may be made by its manufacturer, is not guaranteed or endorsed by the publisher.

## Abbreviations

LCOS: low cardiac output syndrome
PICU: pediatric intensive care unit
AIMS: anesthesia information management system
UMCU: University Medical Center Utrecht
CPB: cardiopulmonary bypass
SD: standard deviation
IQR: interquartile range
PCWP: pulmonary capillary wedge pressure
ECMO: extracorporeal membrane oxygenation
IABP: intra-aortic balloon pump
VAD: ventricle assistant device
CI: cardiac index
VIS: vasoactive-inotropic score
CABG: coronary artery bypass grafting
AV: aortic valve
MV: mitral valve
ASD: atrial septum defect
VSD: ventricle septum defect
PV: pulmonary valve
min: minutes
MNAR: missing not at random
ICU: intensive care unit.
