# Supplementary material for: Definitions of low cardiac output syndrome after cardiac surgery and their effect on the incidence of intraoperative LCOS: A literature review and cohort study
Source: Front Cardiovasc Med. 2022 Sep 29;9:926957. doi: 10.3389/fcvm.2022.926957 (PMC9558721; doi:10.3389/fcvm.2022.926957)
Supplement: Supplementary file 1 [file Data_Sheet_1.docx]

# Supplement 1

# Categorisation of variables of LCOS definition

First the definitions were categorised in ‘children’ or ‘adults’. If the study population had a mean or median age under 18 years old, the study was categorised in ‘children’, if the study population had a mean or median age of 18 years or older, the study was categorised in ‘adults’.

Secondly, we examined if the definitions were reproducible. To be reproducible, definitions needed to have a cut-off value, if the definition included ‘lactate’, ‘saturation’, ‘cardiac index’, ‘high/increasing/maximal inotropic drugs use’, ‘hypotension’, ‘metabolic acidosis’, ‘pulmonary capillary wedge pressure’, ‘cardiac output’, ‘systemic vascular resistance’, ‘fever’, ‘ejection fraction’, ‘oliguria’ or ‘tachycardia’. Furthermore, vague terms were categorised as not reproducible. Vague terms included ‘hemodynamic compromised’, ‘maintain stable hemodynamics’ and clinical signs of hypoperfusion without describing those signs. Definitions did not need a cut-off value to be reproducible for the following clinical signs: ‘altered state of mind’ and ‘cold extremities’ to be categorised as reproducible.

Lastly, we categorised the different parts of the definition. We made no separate categories for variables that occurred in less than 1% of the articles (no. of articles < 3), those were categorised in ‘others’. Variables that occurred in less than 2% of the articles (no. of articles < 5), were categorised as ‘too high’ or ‘too low’ without their cut-off value. Variables that occurred in 2% or more of the articles were also categorised with their cut-off value. After categorizing the definitions, we had thirteen main categories, i.e: ‘inotropes’, ‘mechanical support’, ‘metabolic acidosis’, ‘cardiac function’, ‘blood pressure’, ‘clinical signs of hypoperfusion’, ‘saturation’, ‘pulmonary capillary wedge pressure’, ‘renal replacement therapy’, ‘instable hemodynamics’, cardiac arrest’, ‘death’ and ‘others’.

We found four ways that inotropes were measured and therefor devided inotropes in four subcategories: ‘number of inotropes’, ‘Vasoactive-Inotropes-Score’, ‘duration of inotropes use’ and ‘type of inotropes’.

When a definition included statements like ‘the need for pharmacologic therapy’ or ‘the need for inotropes’, they were categorised in the main category ‘inotropes’ without further specification. If definitions included statements like ‘need for additional inotropes’, they were categorised in the main category ‘inotropes’ and specified as more than one inotrope needed in the subcategory ‘number of inotropes’. If a definition described more than one duration, like ‘the need for inotropes for 24-48 hours’ or ‘the need for norepinephrine for 24 hours or dopamine for 48 hours’, the shortest duration was noted.

The main category ‘mechanical support’ has 3 subcategories: ‘Intra-Aortic Balloon Pump’, ‘Ventricular Assist Device’ and ‘Extracorporeal Membrane Oxygenation’. If a definition included a statement like ‘the need of mechanical support’, without specifying, we categorised them in all three subcategories.

The main category ‘metabolic acidosis’ was devided in four subcategories, ‘high lactate’, ‘high base-deficit’, ‘low pH’ and ‘low bicarbonate’. ‘Lactate’ and ‘base-deficit’ have cut-off values, ‘low pH’ and ‘low bicarbonate’ are only categorised as ‘too low’, because less than 2% of the definitions used these parameters. If definitions included increase in lactate, they were listed as a lactate cut-off value of ‘higher than 2’. If a definition included two kinds of cut-off values (for example “an increase in lactate of 2 or a lactate higher than 4”), they were listed as the lowest value (in this example ‘higher than 2’). If the definition only mentioned a metabolic acidosis, it was listed in the main category without further specifics.

The main category ‘cardiac function’ has three subcategories, ‘cardiac index’, ‘left ventricle ejection fraction’ and ‘low cardiac output’. ‘Cardiac index’ and ‘left ventricle ejection fraction’ were categorised with cut-off values and ‘low cardiac output’ was only dichotomized.

The main category ‘blood pressure’ has four subcategories, called: ‘systolic arterial hypotension’, ‘mean arterial hypotension’, ‘central venous pressure’ and ‘systemic vascular resistance’. Only ‘systemic vascular resistance’ was dichotomized, the other subcategories had cut-off values. If definitions included statements like ‘sank under an age-related normal systolic blood pressure’, they were listed as ‘under p5 systolic blood pressure’. When a definition only mentioned a hypotension without further specifying, they were listed in the main category without further specifics. If a definition mentioned a hypotension under 90mmHg, they were listed in the subcategory ‘systolic arterial hypotension’. When definitions described a range, like ‘a mean arterial pressure under 50-60mmHg’, they were listed with the highest cut-off, in this case they were listed with the cut-off value of 60mmHg.

The main category ‘clinical signs of hypoperfusion’ has six subcategories, named: ‘oliguria’, ‘tachycardia’, ‘cold extremities’, ‘decreased consciousness’, ‘clammy skin’ and ‘other clinical signs of hypoperfusion’. Clinical features that occurred in less than 1% of the articles were categorised in ‘other clinical signs of hypoperfusion’. Definitions including ‘peripheral vasoconstriction’, ‘poor peripheral perfusion’, ‘prolonged capillary refill’ or ‘differences in core-periphery temperature’ were listed as ‘cold extremities’. ‘Oliguria’ and ‘tachycardia’ were the only two subcategories with cut-off values. Definitions including terms like ‘signs of tissue hypoperfusion/organ hypoperfusion’ were listed in the main category ‘clinical signs of hypoperfusion’ without further specifying.

The main category ‘saturation’ has three subcategories, called: ‘differences in arterial and venous saturation’, ‘decreased arterial saturation’ and ‘venous saturation’. ‘Differences in arterial and venous saturation’ and ‘venous saturation’ had cut-off values, ‘decreased arterial saturation’ was only categorised as ‘too low’. From the two subcategories with venous saturations, we also listed if the definition included mixed or central venous saturation.

The main category ‘pulmonary capillary wedge pressure’ has no subcategories, the category was listed with a cut-off value. When definitions described a range, like ‘ a pulmonary capillary wedge pressure between 20-25mmHg’, they were listed with the lowest cut-off value.

The main category ‘renal replacement therapy’, ‘instable hemodynamic’, ‘cardiac arrest’ and ‘death’ were categorised as present or not present.

**Supplement 2**

**Reference list of literature review**

Abella, R., Satriano, A., Frigiola, A., Varrica, A., Gavilanes, A. D., Zimmermann, L. J., Vles, H. J., Florio, P., Calevo, M. G., & Gazzolo, D. (2012). Adrenomedullin alterations related to cardiopulmonary bypass in infants with low cardiac output syndrome. *The Journal of Maternal-Fetal & Neonatal Medicine : The Official Journal of the European Association of Perinatal Medicine, the Federation of Asia and Oceania Perinatal Societies, the International Society of Perinatal Obstetricians*, *25*(12), 2756–2761. https://doi.org/10.3109/14767058.2012.718393

Abrahamov, D., Tamaris, M., Guru, V., Fremes, S., Christakis, G., Bhatnagar, G., Sever, J., & Goldman, B. (1999). Clinical results of endarterectomy of the right and left anterior descending coronary arteries. *Journal of Cardiac Surgery*, *14*(1), 16–25. https://doi.org/10.1111/J.1540-8191.1999.TB00945.X

Açil, T., Türköz, R., Açil, M., Sezgin, A. T., Baltali, M., Gülcan, Ö., Özin, B., & Müderrisoǧlu, H. (2006). Value of prolonged QRS duration as a predictor of low cardiac output syndrome in patients with impaired left ventricular systolic function who undergo isolated coronary artery bypass grafting. *The American Journal of Cardiology*, *98*(10), 1357–1362. https://doi.org/10.1016/J.AMJCARD.2006.06.031

Ak, K., Demirbaş, E., Ataş, H., Birkan, Y., Herz, F. A.-, & 2017, undefined. (n.d.). Results of pericardiectomy for constrictive pericarditis. *Springer*. Retrieved June 23, 2022, from https://link.springer.com/article/10.1007/s00059-016-4436-2

Alexi-Meskhishvili, V., Popov, S. A., & Nikoljuk, A. P. (1984). Evaluation of hemodynamics in infants and small babies after open heart surgery. *The Thoracic and Cardiovascular Surgeon*, *32*(1), 4–9. https://doi.org/10.1055/S-2007-1023335

Algarni, K. D., Maganti, M., & Yau, T. M. (2011). Predictors of low cardiac output syndrome after isolated coronary artery bypass surgery: trends over 20 years. *The Annals of Thoracic Surgery*, *92*(5), 1678–1684. https://doi.org/10.1016/J.ATHORACSUR.2011.06.017

Algarni, K. D., Weisel, R. D., Caldarone, C. A., Maganti, M., Tsang, K., & Yau, T. M. (2013). Microplegia during coronary artery bypass grafting was associated with less low cardiac output syndrome: a propensity-matched comparison. *The Annals of Thoracic Surgery*, *95*(5), 1532–1538. https://doi.org/10.1016/J.ATHORACSUR.2012.09.056

Algarni, K. D., Yanagawa, B., Rao, V., & Yau, T. M. (2014). Profound hypothermia compared with moderate hypothermia in repair of acute type A aortic dissection. *The Journal of Thoracic and Cardiovascular Surgery*, *148*(6), 2888–2894. https://doi.org/10.1016/J.JTCVS.2014.01.020

Amabili, P., Benbouchta, S., Roediger, L., Senard, M., Hubert, M. B., Donneau, A. F., Brichant, J. F., & Hans, G. A. (2018). Low Cardiac Output Syndrome After Adult Cardiac Surgery: Predictive Value of Peak Systolic Global Longitudinal Strain. *Anesthesia and Analgesia*, *126*(5), 1476–1483. https://doi.org/10.1213/ANE.0000000000002605

Amiet, V., Perez, M. H., Longchamp, D., Boulos Ksontini, T., Natterer, J., Plaza Wuthrich, S., Cotting, J., & di Bernardo, S. (2018). Use of Levosimendan in Postoperative Setting After Surgical Repair of Congenital Heart Disease in Children. *Pediatric Cardiology*, *39*(1), 19–25. https://doi.org/10.1007/S00246-017-1718-2

Anantasit, N., Boyd, J. H., Russell, J. A., Fjell, C. D., Lichtenstein, S. v., & Walley, K. R. (2014). Prolonged QTc affects short-term and long-term outcomes in patients with normal left ventricular function undergoing cardiac surgery. *The Journal of Thoracic and Cardiovascular Surgery*, *147*(5), 1627–1633. https://doi.org/10.1016/J.JTCVS.2013.11.043

Ansley, D. M., Raedschelders, K., Chen, D. D. Y., & Choi, P. T. (2009). Rationale, design and baseline characteristics of the PRO-TECT II study: PROpofol CardioproTECTion for Type II diabetics: a randomized, controlled trial of high-dose propofol versus isoflurane preconditioning in patients undergoing on-pump coronary artery bypass graft surgery. *Contemporary Clinical Trials*, *30*(4), 380–385. https://doi.org/10.1016/J.CCT.2009.03.004

Ansley, D. M., Raedschelders, K., Choi, P. T., Wang, B., Cook, R. C., & Chen, D. D. Y. (2016). Propofol cardioprotection for on-pump aortocoronary bypass surgery in patients with type 2 diabetes mellitus (PRO-TECT II): a phase 2 randomized-controlled trial. *Canadian Journal of Anaesthesia = Journal Canadien d’anesthesie*, *63*(4), 442–453. https://doi.org/10.1007/S12630-015-0580-Z

Arafa, O. E., Geiran, O. R., Andersen, K., Fosse, E., Simonsen, S., & Svennevig, J. L. (2000). Intraaortic balloon pumping for predominantly right ventricular failure after heart transplantation. *The Annals of Thoracic Surgery*, *70*(5), 1587–1593. https://doi.org/10.1016/S0003-4975(00)01864-6

Bailey, J. M., Hoffman, T. M., Wessel, D. L., Nelson, D. P., Atz, A. M., Chang, A. C., Kulik, T. J., Spray, T. L., Akbary, A., P.Miller, R., & Wernovsky, G. (2004). A population pharmacokinetic analysis of milrinone in pediatric patients after cardiac surgery. *Journal of Pharmacokinetics and Pharmacodynamics*, *31*(1), 43–59. https://doi.org/10.1023/B:JOPA.0000029488.45177.48

Balderas-Muñoz, K., Rodríguez-Zanella, H., Fritche-Salazar, J. F., Ávila-Vanzzini, N., Juárez Orozco, L. E., Arias-Godínez, J. A., Calvillo-Argüelles, O., Rivera-Peralta, S., Sauza-Sosa, J. C., Ruiz-Esparza, M. E., Bucio-Reta, E., Rómero, A., Espinola-Zavaleta, N., Domínguez-Mendez, B., Gaxiola-Macias, M., & Martínez-Ríos, M. A. (2017). Improving risk assessment for post-surgical low cardiac output syndrome in patients without severely reduced ejection fraction undergoing open aortic valve replacement. The role of global longitudinal strain and right ventricular free wall strain. *The International Journal of Cardiovascular Imaging*, *33*(10), 1483–1489. https://doi.org/10.1007/S10554-017-1139-6

Barile, L., Landoni, G., Pieri, M., Ruggeri, L., Maj, G., Nigro Neto, C., Pasin, L., Cabrini, L., & Zangrillo, A. (2013). Cardiac index assessment by the pressure recording analytic method in critically ill unstable patients after cardiac surgery. *Journal of Cardiothoracic and Vascular Anesthesia*, *27*(6), 1108–1113. https://doi.org/10.1053/J.JVCA.2013.02.016

Beghi, C., Nicolini, F., Budillon, A. M., Borrello, B., Ballore, L., Reverberi, C., & Gherli, T. (2002). Midterm clinical results in myocardial revascularization using the radial artery. *Chest*, *122*(6), 2075–2079. https://doi.org/10.1378/CHEST.122.6.2075

Beiras-Fernandez, A., Kornberger, A., Oberhoffer, M., Kur, F., Weis, M., Vahl, C. F., & Weis, F. (2019). Levosimendan as rescue therapy in low output syndrome after cardiac surgery: effects and predictors of outcome. *The Journal of International Medical Research*, *47*(8), 3502–3512. https://doi.org/10.1177/0300060519835087

Belletti, A., Jacobs, S., Affronti, G., Mladenow, A., Landoni, G., Falk, V., & Schoenrath, F. (2018). Incidence and Predictors of Postoperative Need for High-Dose Inotropic Support in Patients Undergoing Cardiac Surgery for Infective Endocarditis. *Journal of Cardiothoracic and Vascular Anesthesia*, *32*(6), 2528–2536. https://doi.org/10.1053/J.JVCA.2017.12.015

Benedetto, U., Melina, G., Capuano, F., Comito, C., Bianchini, R., Simon, C., Refice, S., Angeloni, E., & Sinatra, R. (2008). Preoperative angiotensin-converting enzyme inhibitors protect myocardium from ischemia during coronary artery bypass graft surgery. *Journal of Cardiovascular Medicine (Hagerstown, Md.)*, *9*(11), 1098–1103. https://doi.org/10.2459/JCM.0B013E32830A6DAF

Biancari, F., Mikkola, R., Heikkinen, J., Lahtinen, J., Kettunen, U., & Juvonen, T. (2012). Individual surgeon’s impact on the risk of re-exploration for excessive bleeding after coronary artery bypass surgery. *Journal of Cardiothoracic and Vascular Anesthesia*, *26*(4), 550–556. https://doi.org/10.1053/J.JVCA.2012.02.009

Biancari, F., Onorati, F., Mariscalco, G., de Feo, M., Messina, A., Santarpino, G., Santini, F., Beghi, C., Ratta, E. della, Troise, G., Fischlein, T., Passerone, G., Juvonen, T., Mazzucco, A., Heikkinen, J., & Faggian, G. (2014). First-time, isolated surgical aortic valve replacement after prior coronary artery bypass surgery: results from the RECORD multicenter registry. *Journal of Cardiac Surgery*, *29*(4), 450–454. https://doi.org/10.1111/JOCS.12365

Boeken, U., Assmann, A., Mehdiani, A., Akhyari, P., & Lichtenberg, A. (2011). Open chest management after cardiac operations: outcome and timing of delayed sternal closure. *European Journal of Cardio-Thoracic Surgery : Official Journal of the European Association for Cardio-Thoracic Surgery*, *40*(5), 1146–1150. https://doi.org/10.1016/J.EJCTS.2011.02.047

Boeken, U., Feindt, P., Schurr, P., Assmann, A., Akhyari, P., & Lichtenberg, A. (2011). Delayed sternal closure (DSC) after cardiac surgery: outcome and prognostic markers. *Journal of Cardiac Surgery*, *26*(1), 22–27. https://doi.org/10.1111/J.1540-8191.2010.01159.X

Böhrer, H., Schmidt, H., Motsch, J., Gust, R., Bach, A., & Martin, E. (1997). Gastric intramucosal pH: a predictor of survival in cardiac surgery patients with low cardiac output? *Journal of Cardiothoracic and Vascular Anesthesia*, *11*(2), 184–186. https://doi.org/10.1016/S1053-0770(97)90211-1

Borger, M. A., Seeburger, J., Walther, T., Borger, F., Rastan, A., Doenst, T., & Mohr, F. W. (2010). Effect of preoperative statin therapy on patients undergoing isolated and combined valvular heart surgery. *The Annals of Thoracic Surgery*, *89*(3), 773–780. https://doi.org/10.1016/J.ATHORACSUR.2009.12.001

Börgermann, J., Hakim, K., Renner, A., Parsa, A., Aboud, A., Becker, T., Masshoff, M., Zittermann, A., Gummert, J. F., & Kuss, O. (2012). Clampless off-pump versus conventional coronary artery revascularization: a propensity score analysis of 788 patients. *Circulation*, *126*(11 Suppl 1). https://doi.org/10.1161/CIRCULATIONAHA.111.084285

Börgermann, J., Lazouski, K., Kuhn, J., Dreier, J., Schmidt, M., Gilis-Januszewski, T., Knabbe, C., Gummert, J. F., & Zittermann, A. (2012). 1,25-Dihydroxyvitamin D fluctuations in cardiac surgery are related to age and clinical outcome*. *Critical Care Medicine*, *40*(7), 2073–2081. https://doi.org/10.1097/CCM.0B013E31824E8C42

Burgos, L. M., Gil Ramírez, A., Seoane, L., Espinoza, J., Furmento, J. F., Costabel, J. P., Benzadón, M., & Navia, D. (2021). Is the Obesity Paradox in Cardiac Surgery Really a Myth? Effect of Body Mass Index on Early and Late Clinical Outcomes. *Journal of Cardiothoracic and Vascular Anesthesia*, *35*(2), 492–498. https://doi.org/10.1053/J.JVCA.2020.03.051

Busro, P. W., Romolo, H., Sastroasmoro, S., Rachmat, J., Sadikin, M., Santoso, A., Boom, C. E., Suwarto, S., & Jusuf, A. A. (2018a). Role of terminal warm blood cardioplegia in complex congenital heart surgery. *Asian Cardiovascular & Thoracic Annals*, *26*(3), 196–202. https://doi.org/10.1177/0218492318759105

Busro, P. W., Romolo, H., Sastroasmoro, S., Rachmat, J., Sadikin, M., Santoso, A., Boom, C. E., Suwarto, S., & Jusuf, A. A. (2018b). Role of terminal warm blood cardioplegia in complex congenital heart surgery. *Asian Cardiovascular & Thoracic Annals*, *26*(3), 196–202. https://doi.org/10.1177/0218492318759105

Butts, R. J., Scheurer, M. A., Atz, A. M., Zyblewski, S. C., Hulsey, T. C., Bradley, S. M., & Graham, E. M. (2012). Comparison of maximum vasoactive inotropic score and low cardiac output syndrome as markers of early postoperative outcomes after neonatal cardiac surgery. *Pediatric Cardiology*, *33*(4), 633–638. https://doi.org/10.1007/S00246-012-0193-Z

Cannesson, M., Bionda, C., Gostoli, B., Raisky, O., di Filippo, S., Bompard, D., Védrinne, C., Rousson, R., Ninet, J., Neidecker, J., & Lehot, J. J. (2007). Time course and prognostic value of plasma B-type natriuretic peptide concentration in neonates undergoing the arterial switch operation. *Anesthesia and Analgesia*, *104*(5), 1059–1065. https://doi.org/10.1213/01.ANE.0000263644.98314.E2

Carmona, F., Manso, P. H., Vicente, W. V. A., Castro, M., & Carlotti, A. P. C. P. (2008). Risk stratification in neonates and infants submitted to cardiac surgery with cardiopulmonary bypass: a multimarker approach combining inflammatory mediators, N-terminal pro-B-type natriuretic peptide and troponin I. *Cytokine*, *42*(3), 317–324. https://doi.org/10.1016/J.CYTO.2008.03.005

Caruba, T., Hourton, D., Sabatier, B., Rousseau, D., Tibi, A., Hoffart-Jourdain, C., Souag, A., Freitas, N., Yjjou, M., Almeida, C., Gomes, N., Aucouturier, P., Djadi-Prat, J., Menasché, P., Chatellier, G., & Cholley, B. (2016). Rationale and design of the multicenter randomized trial investigating the effects of levosimendan pretreatment in patients with low ejection fraction (≤40 %) undergoing CABG with cardiopulmonary bypass (LICORN study). *Journal of Cardiothoracic Surgery*, *11*(1). https://doi.org/10.1186/S13019-016-0530-Z

Cavigelli-Brunner, A., Hug, M. I., Dave, H., Baenziger, O., Buerki, C., Bettex, D., Cannizzaro, V., & Balmer, C. (2018). Prevention of Low Cardiac Output Syndrome After Pediatric Cardiac Surgery: A Double-Blind Randomized Clinical Pilot Study Comparing Dobutamine and Milrinone. *Pediatric Critical Care Medicine : A Journal of the Society of Critical Care Medicine and the World Federation of Pediatric Intensive and Critical Care Societies*, *19*(7), 619–625. https://doi.org/10.1097/PCC.0000000000001533

Chan, K. L., Ip, P., Chiu, C. S. W., & Cheung, Y. F. (2003). Peritoneal dialysis after surgery for congenital heart disease in infants and young children. *The Annals of Thoracic Surgery*, *76*(5), 1443–1449. https://doi.org/10.1016/S0003-4975(03)01026-9

Cholley, B., Caruba, T., Grosjean, S., Amour, J., Ouattara, A., Villacorta, J., Miguet, B., Guinet, P., Lévy, F., Squara, P., Hamou, N. A., Carillon, A., Boyer, J., Boughenou, M. F., Rosier, S., Robin, E., Radutoiu, M., Durand, M., Guidon, C., … Chatellier, G. (2017). Effect of Levosimendan on Low Cardiac Output Syndrome in Patients With Low Ejection Fraction Undergoing Coronary Artery Bypass Grafting With Cardiopulmonary Bypass: The LICORN Randomized Clinical Trial. *JAMA*, *318*(6), 548–556. https://doi.org/10.1001/JAMA.2017.9973

Christenson, J. T., Schmuziger, M., & Simonet, F. (1997). Reoperative coronary artery bypass procedures: risk factors for early mortality and late survival. *European Journal of Cardio-Thoracic Surgery : Official Journal of the European Association for Cardio-Thoracic Surgery*, *11*(1), 129–133. https://doi.org/10.1016/S1010-7940(96)01030-5

Chrysostomou, C., Morell, V. O., Kuch, B. A., O’Malley, E., Munoz, R., & Wearden, P. D. (2013). Short- and intermediate-term survival after extracorporeal membrane oxygenation in children with cardiac disease. *The Journal of Thoracic and Cardiovascular Surgery*, *146*(2), 317–325. https://doi.org/10.1016/J.JTCVS.2012.11.014

Claessens, N. H. P., Chau, V., de Vries, L. S., Jansen, N. J. G., Au-Young, S. H., Stegeman, R., Blaser, S., Shroff, M., Haas, F., Marini, D., Breur, J. M. P. J., Seed, M., Benders, M. J. N. L., & Miller, S. P. (2019). Brain Injury in Infants with Critical Congenital Heart Disease: Insights from Two Clinical Cohorts with Different Practice Approaches. *The Journal of Pediatrics*, *215*, 75-82.e2. https://doi.org/10.1016/J.JPEDS.2019.07.017

Claessens, N. H. P., Jansen, N. J. G., Breur, J. M. P. J., Algra, S. O., Stegeman, R., Alderliesten, T., van Loon, K., de Vries, L. S., Haas, F., Benders, M. J. N. L., & Lemmers, P. M. A. (2019). Postoperative cerebral oxygenation was not associated with new brain injury in infants with congenital heart disease. *The Journal of Thoracic and Cardiovascular Surgery*, *158*(3), 867-877.e1. https://doi.org/10.1016/J.JTCVS.2019.02.106

Coverdale, N. S., Hamilton, A., Petsikas, D., McClure, R. S., Malik, P., Milne, B., Saha, T., Zelt, D., Brown, P., & Payne, D. M. (2018). Remote Ischemic Preconditioning in High-risk Cardiovascular Surgery Patients: A Randomized-controlled Trial. *Seminars in Thoracic and Cardiovascular Surgery*, *30*(1), 26–33. https://doi.org/10.1053/J.SEMTCVS.2017.09.001

Currey, J., & Botti, M. (2005). The haemodynamic status of cardiac surgical patients in the initial 2-h recovery period. *European Journal of Cardiovascular Nursing*, *4*(3), 207–214. https://doi.org/10.1016/J.EJCNURSE.2005.03.007

de Palo, M., Guida, P., Mastro, F., Nanna, D., Quagliara, T. A. P., Rociola, R., Lionetti, G., & Paparella, D. (2017). Myocardial protection during minimally invasive cardiac surgery through right mini-thoracotomy. *Perfusion*, *32*(3), 245–252. https://doi.org/10.1177/0267659116679249

Desai, P. M., Sarkar, M. S., & Umbarkar, S. R. (2018). Prophylactic preoperative levosimendan for off-pump coronary artery bypass grafting in patients with left ventricular dysfunction: Single-centered randomized prospective study. *Annals of Cardiac Anaesthesia*, *21*(2), 123–128. https://doi.org/10.4103/ACA.ACA_178_17

Ding, W. J., Ji, Q., Shi, Y. Q., & Ma, R. H. (2015). Predictors of low cardiac output syndrome after isolated coronary artery bypass grafting. *International Heart Journal*, *56*(2), 144–149. https://doi.org/10.1536/IHJ.14-231

Dolcino, A., Gaudin, R., Pontailler, M., Raisky, O., Vouhé, P., & Bojan, M. (2020). Single-Shot Cold Histidine-Tryptophan-Ketoglutarate Cardioplegia for Long Aortic Cross-Clamping Durations in Neonates. *Journal of Cardiothoracic and Vascular Anesthesia*, *34*(4), 959–965. https://doi.org/10.1053/J.JVCA.2019.08.039

Du, X., Chen, H., Song, X., Wang, S., Hao, Z., Yin, L., & Lu, Z. (2020). Risk factors for low cardiac output syndrome in children with congenital heart disease undergoing cardiac surgery: a retrospective cohort study. *BMC Pediatrics*, *20*(1). https://doi.org/10.1186/S12887-020-1972-Y

Duggal, B., Pratap, U., Slavik, Z., Kaplanova, J., & Macrae, D. (2005). Milrinone and low cardiac output following cardiac surgery in infants: is there a direct myocardial effect? *Pediatric Cardiology*, *26*(5), 642–645. https://doi.org/10.1007/S00246-005-0881-Z

Duncan, A. E., Kartashov, A., Robinson, S. B., Randall, D., Zhang, K., Luber, J., James, R. A., Halvorson, S., & Bokesch, P. (2022). Risk factors, resource use, and cost of postoperative low cardiac output syndrome. *The Journal of Thoracic and Cardiovascular Surgery*, *163*(5), 1890-1898.e10. https://doi.org/10.1016/J.JTCVS.2020.06.125

Dupuis, J. Y., Bondy, R., Cattran, C., Nathan, H. J., & Earl Wynands, J. (1992). Amrinone and dobutamine as primary treatment of low cardiac output syndrome following coronary artery surgery: a comparison of their effects on hemodynamics and outcome. *Journal of Cardiothoracic and Vascular Anesthesia*, *6*(5), 542–553. https://doi.org/10.1016/1053-0770(92)90096-P

Ellenberger, C., Sologashvili, T., Cikirikcioglu, M., Verdon, G., Diaper, J., Cassina, T., & Licker, M. (2017). Risk factors of postcardiotomy ventricular dysfunction in moderate-to-high risk patients undergoing open-heart surgery. *Annals of Cardiac Anaesthesia*, *20*(3), 287–296. https://doi.org/10.4103/ACA.ACA_60_17

Ellenberger, C., Sologashvili, T., Kreienbühl, L., Cikirikcioglu, M., Diaper, J., & Licker, M. (2018). Myocardial Protection by Glucose-Insulin-Potassium in Moderate- to High-Risk Patients Undergoing Elective On-Pump Cardiac Surgery: A Randomized Controlled Trial. *Anesthesia and Analgesia*, *126*(4), 1133–1141. https://doi.org/10.1213/ANE.0000000000002777

Ergün, S., Yildiz, O., Güneş, M., Akdeniz, H. S., Öztürk, E., Onan, İ. S., Güzeltaş, A., & Haydin, S. (2020). Use of extracorporeal membrane oxygenation in postcardiotomy pediatric patients: parameters affecting survival. *Perfusion*, *35*(7), 608–620. https://doi.org/10.1177/0267659119897746

Erkut, B., Dag, O., Kaygin, M. A., Senocak, M., Limandal, H. K., Arslan, U., Kiymaz, A., Aydin, A., Kahraman, N., & Calik, E. S. (2013). On-pump beating-heart versus conventional coronary artery bypass grafting for revascularization in patients with severe left ventricular dysfunction: early outcomes. *Canadian Journal of Surgery. Journal Canadien de Chirurgie*, *56*(6), 398–404. https://doi.org/10.1503/CJS.018412

Fathi, M., Valaei, M., Ghanbari, A., Ghasemi, R., & Yaghubi, M. (2020). Comparison of Patient’s Kidney Function Based on Kidney Disease Improving Global Outcomes (KDIGO) Criteria and Clinical Parameters in Isolated Coronary Artery Bypass Graft (CABG) Surgery in On-Pump and Off-pump Methods in Patients with Low Cardiac Output Syndrome (LCOS) After Surgery. *Anesthesiology and Pain Medicine*, *10*(2). https://doi.org/10.5812/AAPM.100517

Favia, I., Rizza, A., Garisto, C., Haiberger, R., di Chiara, L., Romagnoli, S., & Ricci, Z. (2016). Cardiac index assessment by the pressure recording analytical method in infants after paediatric cardiac surgery: a pilot retrospective study. *Interactive Cardiovascular and Thoracic Surgery*, *23*(6), 919–923. https://doi.org/10.1093/ICVTS/IVW251

Fawzy, H. F., Morsy, A. A., Serag, A. R., Elkahwagy, M. S., Sami, G., Wahby, E. A., & Arafat, A. A. (2020). Should Moderate Functional Tricuspid Regurgitation Be Repaired During Surgery for Rheumatic Mitral Valve Disease? *Heart, Lung & Circulation*, *29*(10), 1554–1560. https://doi.org/10.1016/J.HLC.2020.01.014

Flores, S., Cooper, D. S., Opoka, A. M., Iliopoulos, I., Pluckebaum, S., Alder, M. N., Krallman, K. A., Sahay, R. D., Fei, L., & Wong, H. R. (2018). Characterization of the Glucocorticoid Receptor in Children Undergoing Cardiac Surgery. *Pediatric Critical Care Medicine : A Journal of the Society of Critical Care Medicine and the World Federation of Pediatric Intensive and Critical Care Societies*, *19*(8), 705–712. https://doi.org/10.1097/PCC.0000000000001572

Flores, S., Fitzgerald, M. R., Iliopoulos, I., Daily, J. A., Rodriguez, M., Nelson, D. P., Wong, H. R., Menon, K., & Cooper, D. S. (2017). An International Survey of Corticosteroid Use for the Management of Low Cardiac Output Syndrome. *Pediatric Critical Care Medicine : A Journal of the Society of Critical Care Medicine and the World Federation of Pediatric Intensive and Critical Care Societies*, *18*(7), 630–637. https://doi.org/10.1097/PCC.0000000000001180

Formica, F., Mariani, S., D’Alessandro, S., Singh, G., di Mauro, M., Cerrito, M. G., Messina, L. A., Scianna, S., Papesso, F., & Sangalli, F. (2020). Does additional coronary artery bypass grafting to aortic valve replacement in elderly patients affect the early and long-term outcome? *Heart and Vessels*, *35*(4), 487–501. https://doi.org/10.1007/S00380-019-01519-6

Foulks, M. G., Meyer, R. M. L., Gold, J. I., Herrington, C. S., Kallin, K., & Menteer, J. D. (2019). Postoperative heart failure after stage 1 palliative surgery for single ventricle cardiac disease. *Pediatric Cardiology*, *40*(5), 943–949. https://doi.org/10.1007/S00246-019-02093-4

Fuster, R. G., Argudo, J. A. M., Albarova, O. G., Sos, F. H., López, S. C., Sorlí, M. J. D., Codoñer, M. B., & Miñano, J. A. B. (2003). Left ventricular mass index in aortic valve surgery: a new index for early valve replacement? *European Journal of Cardio-Thoracic Surgery : Official Journal of the European Association for Cardio-Thoracic Surgery*, *23*(5), 696–702. https://doi.org/10.1016/S1010-7940(03)00092-7

Garcia Guerra, G., Joffe, A. R., Senthilselvan, A., Kutsogiannis, D. J., & Parshuram, C. S. (2013). Incidence of milrinone blood levels outside the therapeutic range and their relevance in children after cardiac surgery for congenital heart disease. *Intensive Care Medicine*, *39*(5), 951–957. https://doi.org/10.1007/S00134-013-2858-3

Garcia Guerra, G., Robertson, C. M. T., Alton, G. Y., Joffe, A. R., Dinu, I. A., Nicholas, D., Ross, D. B., & Rebeyka, I. M. (2013). Quality of life 4 years after complex heart surgery in infancy. *The Journal of Thoracic and Cardiovascular Surgery*, *145*(2). https://doi.org/10.1016/J.JTCVS.2012.03.050

García-Fuster, R., Estevez, V., Gil, O., Cánovas, S., & Martínez-Leon, J. (2008). Mitral valve replacement in rheumatic patients: effects of chordal preservation. *The Annals of Thoracic Surgery*, *86*(2), 472–481. https://doi.org/10.1016/J.ATHORACSUR.2008.04.046

Gaszewska-Żurek, E., Żurek, P., Kaźmierski, M., Kargul, T., Duraj, P., Jasiński, M., Woś, S., & Tendera, M. (2009). Coronary artery bypass grafting in patients with relatively recent previous stent implantation: three years follow-up results. *Journals.Viamedica.Pl*, *16*(4), 312–316. https://journals.viamedica.pl/cardiology_journal/article/view/21476

George, M., Lehot, J. J., Bastien, O., Durand, P. G., & Estanove, S. (1989). Comparison of cardiovascular effects of dobutamine and enoximone in treatment of low cardiac output syndrome after valvular surgery--preliminary results. *Journal of Cardiothoracic Anesthesia*, *3*(5 Suppl 1), 12. https://doi.org/10.1016/0888-6296(89)90755-2

Gist, K. M., Goldstein, S. L., Joy, M. S., & Vinks, A. A. (2016). Milrinone Dosing Issues in Critically Ill Children With Kidney Injury: A Review. *Journal of Cardiovascular Pharmacology*, *67*(2), 175–181. https://doi.org/10.1097/FJC.0000000000000327

Graham, E. M., Atz, A. M., Butts, R. J., Baker, N. L., Zyblewski, S. C., Deardorff, R. L., Desantis, S. M., Reeves, S. T., Bradley, S. M., & Spinale, F. G. (2011). Standardized preoperative corticosteroid treatment in neonates undergoing cardiac surgery: results from a randomized trial. *The Journal of Thoracic and Cardiovascular Surgery*, *142*(6), 1523–1529. https://doi.org/10.1016/J.JTCVS.2011.04.019

Grieshaber, P., Roth, P., Oster, L., Schneider, T. M., Görlach, G., Nieman, B., & Böning, A. (2017). Is delayed surgical revascularization in acute myocardial infarction useful or dangerous? New insights into an old problem. *Interactive Cardiovascular and Thoracic Surgery*, *25*(5), 772–779. https://doi.org/10.1093/ICVTS/IVX188

Griffith, B. P., Anderson, M. B., Samuels, L. E., Pae, W. E., Naka, Y., & Frazier, O. H. (2013). The RECOVER I: a multicenter prospective study of Impella 5.0/LD for postcardiotomy circulatory support. *The Journal of Thoracic and Cardiovascular Surgery*, *145*(2), 548–554. https://doi.org/10.1016/J.JTCVS.2012.01.067

Guerrero Orriach, J. L., Navarro Arce, I., Hernandez Rodriguez, P., Raigón Ponferrada, A., Malo Manso, A., Ramirez Aliaga, M., Ramirez Fernandez, A., Escalona Belmonte, J. J., Bellido Estevez, I., Gomez Luque, A., Barrera Serrano, R., Toledo Medina, C. S., Rubio Navarro, M., & Cruz Mañas, J. (2019). Preservation of renal function in cardiac surgery patients with low cardiac output syndrome: levosimendan vs beta agonists. *BMC Anesthesiology*, *19*(1). https://doi.org/10.1186/S12871-019-0888-2

Guo, Y., He, S., Wang, T., Chen, Z., & Shu, Y. (2019). Comparison of modified total leaflet preservation, posterior leaflet preservation, and no leaflet preservation techniques in mitral valve replacement - a retrospective study. *Journal of Cardiothoracic Surgery*, *14*(1). https://doi.org/10.1186/S13019-019-0918-7

Hassinger, A. B., Wainwright, M. S., Lane, J. C., Haymond, S., Backer, C. L., & Wald, E. (2012). Elevated preoperative serum asymmetrical dimethylarginine (ADMA) is associated with poor outcomes after pediatric cardiac surgery. *Intensive Care Medicine*, *38*(10), 1697–1704. https://doi.org/10.1007/S00134-012-2657-2

Heinze, H., Heringlake, M., Schmucker, P., & Uhlig, T. (2006). Effects of intra-aortic balloon counterpulsation on parameters of tissue oxygenation. *European Journal of Anaesthesiology*, *23*(7), 555–562. https://doi.org/10.1017/S0265021505001973

Hickok, R. L., Spaeder, M. C., Berger, J. T., Schuette, J. J., & Klugman, D. (2016). Postoperative Abdominal NIRS Values Predict Low Cardiac Output Syndrome in Neonates. *World Journal for Pediatric & Congenital Heart Surgery*, *7*(2), 180–184. https://doi.org/10.1177/2150135115618939

Hisatomi, K., Isomura, T., & Aoyagi, S. (1996). Postoperative changes in myosin light chain and comparison with postoperative clinical variables in patients who have undergone valve surgery. *The Thoracic and Cardiovascular Surgeon*, *44*(6), 296–299. https://doi.org/10.1055/S-2007-1012040

Hoda, M., Haque, A., Aijaz, F., Akhtar, M. I., Rehmat, A., Amanullah, M., & Hasan, B. S. (2016). On-table Extubation after Open Heart Surgery in Children: An Experience from a Tertiary Care Hospital in a Developing Country. *Congenital Heart Disease*, *11*(1), 58–62. https://doi.org/10.1111/CHD.12277

Hoefer, D., Ruttmann, E., Riha, M., Schobersberger, W., Mayr, A., Laufer, G., & Bonatti, J. (2002). Factors influencing intensive care unit length of stay after surgery for acute aortic dissection type A. *The Annals of Thoracic Surgery*, *73*(3), 714–718. https://doi.org/10.1016/S0003-4975(01)03572-X

Hoffman, T. M., Wemovsky, G., Atz, A. M., Kulik, T. J., Nelson, D. P., Chang, A. C., Bailey, J. M., Akbary, A., Kocsis, J. F., Kaczmarek, R., Spray, T. L., & Wessel, D. L. (2003). Efficacy and safety of milrinone in preventing low cardiac output syndrome in infants and children after corrective surgery for congenital heart disease. *Circulation*, *107*(7), 996–1002. https://doi.org/10.1161/01.CIR.0000051365.81920.28

Hoffman, T. M., Wernovsky, G., Atz, A. M., Bailey, J. M., Akbary, A., Kocsis, J. F., Nelson, D. P., Chang, A. C., Kulik, T. J., Spray, T. L., & Wessel, D. L. (2002). Prophylactic intravenous use of milrinone after cardiac operation in pediatrics (PRIMACORP) study. Prophylactic Intravenous Use of Milrinone After Cardiac Operation in Pediatrics. *American Heart Journal*, *143*(1), 15–21. https://doi.org/10.1067/MHJ.2002.120305

Hogue, C. W., de Wet, C. J., Schechtman, K. B., & Dávila-Román, V. G. (2003). The importance of prior stroke for the adjusted risk of neurologic injury after cardiac surgery for women and men. *Anesthesiology*, *98*(4), 823–829. https://doi.org/10.1097/00000542-200304000-00006

Hogue, C. W., Murphy, S. F., Schechtman, K. B., & Dávila-Román, V. G. (1999). Risk factors for early or delayed stroke after cardiac surgery. *Circulation*, *100*(6), 642–647. https://doi.org/10.1161/01.CIR.100.6.642

Hogue, C. W., Palin, C. A., Kailasam, R., Lawton, J. S., Nassief, A., Dávila-Román, V. G., Thomas, B., & Damiano, R. (2006). C-reactive protein levels and atrial fibrillation after cardiac surgery in women. *The Annals of Thoracic Surgery*, *82*(1), 97–102. https://doi.org/10.1016/J.ATHORACSUR.2006.02.043

Hogue, C. W., Sundt, T., Barzilai, B., Schecthman, K. B., & Dávila-Román, V. G. (2001). Cardiac and neurologic complications identify risks for mortality for both men and women undergoing coronary artery bypass graft surgery. *Anesthesiology*, *95*(5), 1074–1078. https://doi.org/10.1097/00000542-200111000-00008

Hori, D., Ono, M., Rappold, T. E., Conte, J. v., Shah, A. S., Cameron, D. E., Adachi, H., Everett, A. D., & Hogue, C. W. (2015). Hypotension After Cardiac Operations Based on Autoregulation Monitoring Leads to Brain Cellular Injury. *The Annals of Thoracic Surgery*, *100*(2), 487–493. https://doi.org/10.1016/J.ATHORACSUR.2015.03.036

Howell, N. J., Ashrafian, H., Drury, N. E., Ranasinghe, A. M., Contractor, H., Isackson, H., Calvert, M., Williams, L. K., Freemantle, N., Quinn, D. W., Green, D., Frenneaux, M., Bonser, R. S., Mascaro, J. G., Graham, T. R., Rooney, S. J., Wilson, I. C., & Pagano, D. (2011). Glucose-insulin-potassium reduces the incidence of low cardiac output episodes after aortic valve replacement for aortic stenosis in patients with left ventricular hypertrophy: results from the Hypertrophy, Insulin, Glucose, and Electrolytes (HINGE) trial. *Circulation*, *123*(2), 170–177. https://doi.org/10.1161/CIRCULATIONAHA.110.945170

Hsu, J. H., Keller, R. L., Chikovani, O., Cheng, H., Hollander, S. A., Karl, T. R., Azakie, A., Adatia, I., Oishi, P., & Fineman, J. R. (2007). B-type natriuretic peptide levels predict outcome after neonatal cardiac surgery. *The Journal of Thoracic and Cardiovascular Surgery*, *134*(4), 939–945. https://doi.org/10.1016/J.JTCVS.2007.04.017

Hummel, J., Rücker, G., & Stiller, B. (2017a). Prophylactic levosimendan for the prevention of low cardiac output syndrome and mortality in paediatric patients undergoing surgery for congenital heart disease. *The Cochrane Database of Systematic Reviews*, *3*(3). https://doi.org/10.1002/14651858.CD011312.PUB2

Hummel, J., Rücker, G., & Stiller, B. (2017b). Prophylactic levosimendan for the prevention of low cardiac output syndrome and mortality in paediatric patients undergoing surgery for congenital heart disease. *The Cochrane Database of Systematic Reviews*, *8*(8). https://doi.org/10.1002/14651858.CD011312.PUB3

Iliopoulos, I., Alder, M. N., Cooper, D. S., Villarreal, E. G., Loomba, R., Sahay, R. D., Fei, L., Steele, P. E., & Flores, S. (2020). Pre-operative neutrophil-lymphocyte ratio predicts low cardiac output in children after cardiac surgery. *Cardiology in the Young*, *30*(4). https://doi.org/10.1017/S1047951120000487

Ivanov, J., Borger, M. A., Rao, V., & David, T. E. (2006). The Toronto Risk Score for adverse events following cardiac surgery. *The Canadian Journal of Cardiology*, *22*(3), 221–227. https://doi.org/10.1016/S0828-282X(06)70900-X

James, C., Millar, J., Horton, S., Brizard, C., Molesworth, C., & Butt, W. (2016). Nitric oxide administration during paediatric cardiopulmonary bypass: a randomised controlled trial. *Intensive Care Medicine*, *42*(11), 1744–1752. https://doi.org/10.1007/S00134-016-4420-6

Jeganathan, R., Armstrong, S., Al-Alao, B., & David, T. (2013). The risk and outcomes of reoperative tricuspid valve surgery. *The Annals of Thoracic Surgery*, *95*(1), 119–124. https://doi.org/10.1016/J.ATHORACSUR.2012.08.058

Jha, A. K., & Hittalmani, S. K. (2017). Septic Shock in Low-Cardiac-Output Patients With Heart and Lung Transplantation: Diagnosis and Management Dilemma. *Journal of Cardiothoracic and Vascular Anesthesia*, *31*(4), 1389–1396. https://doi.org/10.1053/J.JVCA.2016.11.003

Jouan, J., Golmard, L., Benhamouda, N., Durrleman, N., Golmard, J. L., Ceccaldi, R., Trinquart, L., Fabiani, J. N., Tartour, E., Jeunemaitre, X., & Menasché, P. (2012). Gene polymorphisms and cytokine plasma levels as predictive factors of complications after cardiopulmonary bypass. *The Journal of Thoracic and Cardiovascular Surgery*, *144*(2), 467-473.e2. https://doi.org/10.1016/J.JTCVS.2011.12.022

Jung, W., Choi, J. W., Hwang, H. Y., & Kim, K. H. (2018). Early Clinical Outcomes of Tricuspid Valve Repair with a Tri-Ad Annuloplasty Ring in Comparison with the Outcomes Using an MC 3 Ring. *The Korean Journal of Thoracic and Cardiovascular Surgery*, *51*(2), 92–99. https://doi.org/10.5090/KJTCS.2018.51.2.92

K. Chandler, H., & Kirsch, R. (2016). Management of the Low Cardiac Output Syndrome Following Surgery for Congenital Heart Disease. *Current Cardiology Reviews*, *12*(2), 107–111. https://doi.org/10.2174/1573403X12666151119164647

Kavanagh, B. P., Mazer, C. D., Panos, A., & Lichtenstein, S. v. (1992). Effect of warm heart surgery on perioperative management of patients undergoing urgent cardiac surgery. *Journal of Cardiothoracic and Vascular Anesthesia*, *6*(2), 127–131. https://doi.org/10.1016/1053-0770(92)90185-A

Killen, D. A., Piehler, J. M., Borkon, A. M., Gorton, M. E., & Reed, W. A. (1997). Early repair of postinfarction ventricular septal rupture. *The Annals of Thoracic Surgery*, *63*(1), 138–142. https://doi.org/10.1016/S0003-4975(96)00765-5

Killen, D. A., Reed, W. A., Wathanacharoen, S., Beauchamp, G., & Rutherford, B. (1983). Surgical treatment of papillary muscle rupture. *The Annals of Thoracic Surgery*, *35*(3), 243–248. https://doi.org/10.1016/S0003-4975(10)61551-2

Kim, B. J., Kim, Y. S., Kim, H. J., Ju, M. H., Kim, J. B., Jung, S. H., Choo, S. J., & Chung, C. H. (2018). Concomitant mitral valve surgery in patients with moderate ischemic mitral regurgitation undergoing coronary artery bypass grafting. *Journal of Thoracic Disease*, *10*(6), 3632–3642. https://doi.org/10.21037/JTD.2018.05.148

Kim, H. seon, Kim, K. B., Hwang, H. Y., Chang, H. W., & Park, K. J. (2012). Subxiphoid incisional hernia development after coronary artery bypass grafting. *The Korean Journal of Thoracic and Cardiovascular Surgery*, *45*(3), 161–165. https://doi.org/10.5090/KJTCS.2012.45.3.161

Kim, J. S., Jeong, J. H., Moon, S. J., Ahn, H., & Hwang, H. Y. (2016). Sufficient myocardial protection of del Nido cardioplegia regardless of ventricular mass and myocardial ischemic time in adult cardiac surgical patients. *Journal of Thoracic Disease*, *8*(8), 2004–2010. https://doi.org/10.21037/JTD.2016.06.66

Klotz, S., Rukosujew, A., Welp, H., Schmid, C., Tjan, T. D. T., & Scheld, H. H. (2007). Primary extracorporeal membrane oxygenation versus primary ventricular assist device implantation in low cardiac output syndrome following cardiac operation. *Artificial Organs*, *31*(5), 390–394. https://doi.org/10.1111/J.1525-1594.2007.00397.X

Koster, A., Zittermann, A., Börgermann, J., Knabbe, C., Diekmann, J., Schirmer, U., & Gummert, J. F. (2016). Transfusion of 1 and 2 units of red blood cells does not increase mortality and organ failure in patients undergoing isolated coronary artery bypass grafting. *European Journal of Cardio-Thoracic Surgery : Official Journal of the European Association for Cardio-Thoracic Surgery*, *49*(3), 931–936. https://doi.org/10.1093/EJCTS/EZV252

Kulik, A., Rubens, F. D., Gunning, D., Bourke, M. E., Mesana, T. G., & Ruel, M. (2007). Radial artery graft treatment with phenoxybenzamine is clinically safe and may reduce perioperative myocardial injury. *The Annals of Thoracic Surgery*, *83*(2), 502–509. https://doi.org/10.1016/J.ATHORACSUR.2006.09.089

Kumar, A., Puri, G. D., & Bahl, A. (2017). Transesophageal Echocardiography, 3-Dimensional and Speckle Tracking Together as Sensitive Markers for Early Outcome in Patients With Left Ventricular Dysfunction Undergoing Cardiac Surgery. *Journal of Cardiothoracic and Vascular Anesthesia*, *31*(5), 1695–1701. https://doi.org/10.1053/J.JVCA.2017.04.006

Kumon, K., Tanaka, K., Nakajima, N., Naito, Y., & Fujita, T. (1985). Pulmonary circulation in low cardiac output syndrome following open heart surgery. *Japanese Circulation Journal*, *49*(10), 1055–1062. https://doi.org/10.1253/JCJ.49.1055

Kumon, K., Tanaka, K., Rata, T. H., Naito, Y., & Fujita, T. (1986). Organ failures due to low cardiac output syndrome following open heart surgery. *Japanese Circulation Journal*, *50*(4), 329–335. https://doi.org/10.1253/JCJ.50.329

Kunt, A. S., & Andac, M. H. (2012). Decrease of total antioxidative capacity in developed low cardiac output syndrome. *Oxidative Medicine and Cellular Longevity*, *2012*. https://doi.org/10.1155/2012/356301

Kupferschmid, J. P., Rosengart, T. K., McIntosh, C. L., Leon, M. B., & Clark, R. E. (1989). Amiodarone-induced complications after cardiac operation for obstructive hypertrophic cardiomyopathy. *The Annals of Thoracic Surgery*, *48*(3), 359–364. https://doi.org/10.1016/S0003-4975(10)62857-3

Kurobe, H., Kitaichi, T., Shimahara, Y., Kanemura, T., Kanbara, T., Kurushima, A., Kano, M., Hori, T., Yoshida, H., Urata, M., & Kitagawa, T. (2007). Significance of peritoneal fluid drainage in management after repair of complex heart defects in infancy: cytokine dynamics in vivo. *Circulation Journal : Official Journal of the Japanese Circulation Society*, *71*(6), 941–947. https://doi.org/10.1253/CIRCJ.71.941

Lahtinen, J., Biancari, F., Ala-Kokko, T., Rainio, P., Salmela, E., Pokela, R., Satta, J., Lepojärvi, M., & Juvonen, T. (2004). Pulmonary artery blood temperature at admission to the intensive care unit is predictive of outcome after on-pump coronary artery bypass surgery. *Scandinavian Cardiovascular Journal : SCJ*, *38*(2), 104–112. https://doi.org/10.1080/14017430410028500

Leacche, M., Byrne, J. G., Solenkova, N. S., Reagan, B., Mohamed, T. I., Fredi, J. L., & Zhao, D. X. (2013). Comparison of 30-day outcomes of coronary artery bypass grafting surgery verus hybrid coronary revascularization stratified by SYNTAX and euroSCORE. *The Journal of Thoracic and Cardiovascular Surgery*, *145*(4), 1004–1012. https://doi.org/10.1016/J.JTCVS.2012.03.062

Leacche, M., Carrier, M., Bouchard, D., … M. P.-H. S., & 2003, undefined. (2003). Improving neurologic outcome in off-pump surgery: the “no touch” technique. *Researchgate.Net*. https://www.researchgate.net/profile/Denis-Bouchard-2/publication/10696150_Improving_neurologic_outcome_in_off-pump_surgery_The_no_touch_technique/links/54117a0e0cf2b4da1bec4e67/Improving-neurologic-outcome-in-off-pump-surgery-The-no-touch-technique.pdf

Lechner, E., Hofer, A., Leitner-Peneder, G., Freynschlag, R., Mair, R., Weinzettel, R., Rehak, P., & Gombotz, H. (2012). Levosimendan versus milrinone in neonates and infants after corrective open-heart surgery: a pilot study. *Pediatric Critical Care Medicine : A Journal of the Society of Critical Care Medicine and the World Federation of Pediatric Intensive and Critical Care Societies*, *13*(5), 542–548. https://doi.org/10.1097/PCC.0B013E3182455571

Lee, C. H., Ju, M. H., Kim, J. B., Chung, C. H., Jung, S. H., Choo, S. J., & Lee, J. W. (2014). Myocardial injury following aortic valve replacement for severe aortic stenosis: risk factor of postoperative myocardial injury and its impact on long-term outcomes. *The Korean Journal of Thoracic and Cardiovascular Surgery*, *47*(3), 233–239. https://doi.org/10.5090/KJTCS.2014.47.3.233

Lee, W., Kim, J. B., Yang, D. H., Kim, C., Kim, J., Ju, M. H., Kim, H. J., Kang, J. W., Jung, S. H., Kim, Y. H., Choo, S. J., Lee, C. W., Chung, C. H., Lee, J. W., & Lim, T. H. (2018). Comparative effectiveness of coronary screening in heart valve surgery: Computed tomography versus conventional coronary angiography. *The Journal of Thoracic and Cardiovascular Surgery*, *155*(4), 1423-1431.e3. https://doi.org/10.1016/J.JTCVS.2017.10.128

Lee, W. Y., Yoo, J. S., Kim, J. B., Jung, S. H., Choo, S. J., Chung, C. H., & Lee, J. W. (2014). Outcomes of open surgical repair of descending thoracic aortic disease. *The Korean Journal of Thoracic and Cardiovascular Surgery*, *47*(3), 255–261. https://doi.org/10.5090/KJTCS.2014.47.3.255

Levin, R., Degrange, M., del Mazo, C., Tanus, E., & Porcile, R. (2012). Preoperative levosimendan decreases mortality and the development of low cardiac output in high-risk patients with severe left ventricular dysfunction. *Exp Clin Cardiol*, *17*. https://noticias.uai.edu.ar/blogs/2001-3000/2847-InformeMedicina.pdf

Lex, D. J., Tóth, R., Czobor, N. R., Alexander, S. I., Breuer, T., Sápi, E., Szatmári, A., Székely, E., Gál, J., & Székely, A. (2016). Fluid Overload Is Associated With Higher Mortality and Morbidity in Pediatric Patients Undergoing Cardiac Surgery. *Pediatric Critical Care Medicine : A Journal of the Society of Critical Care Medicine and the World Federation of Pediatric Intensive and Critical Care Societies*, *17*(4), 307–314. https://doi.org/10.1097/PCC.0000000000000659

Lin, J. J., Banwell, B. L., Berg, R. A., Dlugos, D. J., Ichord, R. N., Kilbaugh, T. J., Kirsch, R. E., Kirschen, M. P., Licht, D. J., Massey, S. L., Naim, M. Y., Rintoul, N. E., Topjian, A. A., & Abend, N. S. (2017). Electrographic Seizures in Children and Neonates Undergoing Extracorporeal Membrane Oxygenation. *Pediatric Critical Care Medicine : A Journal of the Society of Critical Care Medicine and the World Federation of Pediatric Intensive and Critical Care Societies*, *18*(3), 249–257. https://doi.org/10.1097/PCC.0000000000001067

Lio, A., Bovio, E., Nicolò, F., Saitto, G., Scafuri, A., Bassano, C., Chiariello, L., & Ruvolo, G. (2019). Influence of Body Mass Index on Outcomes of Patients Undergoing Surgery for Acute Aortic Dissection: A Propensity-Matched Analysis. *Texas Heart Institute Journal*, *46*(1), 7–13. https://doi.org/10.14503/THIJ-17-6365

Loforte, A., Walter, E. M. D., Stiller, B., Huebler, M., Alexi-Meskishvili, V., Boettcher, W., Berger, F., & Hetzer, R. (2010). Extracorporeal membrane oxygenation for intraoperative cardiac support in children with congenital heart disease. *Interactive Cardiovascular and Thoracic Surgery*, *10*(5), 753–758. https://doi.org/10.1510/ICVTS.2009.220475

Loskutov, O., Maruniak, S., Dryzhyna, O., Malysh, I., Kolesnykov, V., & Korotchuk, N. (2020). Influence of low-opioid anesthesia in cardiac surgery on dynamics of pro-inflammatory interleukin-6. *Kardiochirurgia i Torakochirurgia Polska = Polish Journal of Cardio-Thoracic Surgery*, *17*(1), 39–43. https://doi.org/10.5114/KITP.2020.94190

Maganti, M., Badiwala, M., Sheikh, A., Scully, H., Feindel, C., David, T. E., & Rao, V. (2010). Predictors of low cardiac output syndrome after isolated mitral valve surgery. *The Journal of Thoracic and Cardiovascular Surgery*, *140*(4), 790–796. https://doi.org/10.1016/J.JTCVS.2009.11.022

Maganti, M., Brister, S. J., Yau, T. M., Collins, S., Badiwala, M., & Rao, V. (2011). Changing trends in emergency coronary bypass surgery. *The Journal of Thoracic and Cardiovascular Surgery*, *142*(4), 816–822. https://doi.org/10.1016/J.JTCVS.2011.01.021

Maganti, M. D., Rao, V., Borger, M. A., Ivanov, J., & David, T. E. (2005). Predictors of low cardiac output syndrome after isolated aortic valve surgery. *Circulation*, *112*(9 Suppl). https://doi.org/10.1161/CIRCULATIONAHA.104.526087

Manso, P. H., Carmona, F., Dal-Pizzol, F., Petronilho, F., Cardoso, F., Castro, M., & Carlotti, A. P. C. P. (2013). Oxidative stress markers are not associated with outcomes after pediatric heart surgery. *Paediatric Anaesthesia*, *23*(2), 188–194. https://doi.org/10.1111/PAN.12040

Martens, A., Beckmann, E., Kaufeld, T., Umminger, J., Fleissner, F., Koigeldiyev, N., Krueger, H., Puntigam, J., Haverich, A., & Shrestha, M. (2016). Total aortic arch repair: risk factor analysis and follow-up in 199 patients. *European Journal of Cardio-Thoracic Surgery : Official Journal of the European Association for Cardio-Thoracic Surgery*, *50*(5), 940–948. https://doi.org/10.1093/EJCTS/EZW158

Martens, A., Koigeldiyev, N., Beckmann, E., Fleissner, F., Kaufeld, T., Krueger, H., Stanelle, D., Puntigam, J., Haverich, A., & Shrestha, M. (2016). Do not leave the heart arrested. Non-cardioplegic continuous myocardial perfusion during complex aortic arch repair improves cardiac outcome. *European Journal of Cardio-Thoracic Surgery : Official Journal of the European Association for Cardio-Thoracic Surgery*, *49*(1), 141–148. https://doi.org/10.1093/EJCTS/EZV009

Martin, K., Breuer, T., Gertler, R., Hapfelmeier, A., Schreiber, C., Lange, R., Hess, J., & Wiesner, G. (2011). Tranexamic acid versus ɛ-aminocaproic acid: efficacy and safety in paediatric cardiac surgery. *European Journal of Cardio-Thoracic Surgery : Official Journal of the European Association for Cardio-Thoracic Surgery*, *39*(6), 892–897. https://doi.org/10.1016/J.EJCTS.2010.09.041

Marwali, E. M., Caesa, P., Darmaputri, S., Sani, A. A., Roebiono, P. S., Fakhri, D., Djer, M. M., Munasir, Z. M., Batubara, J. R. L., Satroasmoro, S., Portman, M. A., & Haas, N. A. (2019). Oral Triiodothyronine Supplementation Decreases Low Cardiac Output Syndrome After Pediatric Cardiac Surgery. *Pediatric Cardiology*, *40*(6), 1238–1246. https://doi.org/10.1007/S00246-019-02143-X

Matsuda, S., Fukui, T., Shimizu, J., Takao, A., Takanashi, S., & Tomoike, H. (2013). Associations between preoperative anemia and outcomes after off-pump coronary artery bypass grafting. *The Annals of Thoracic Surgery*, *95*(3), 854–860. https://doi.org/10.1016/J.ATHORACSUR.2012.10.005

Mehta, R. H., Leimberger, J. D., van Diepen, S., Meza, J., Wang, A., Jankowich, R., Harrison, R. W., Hay, D., Fremes, S., Duncan, A., Soltesz, E. G., Luber, J., Park, S., Argenziano, M., Murphy, E., Marcel, R., Kalavrouziotis, D., Nagpal, D., Bozinovski, J., … Alexander, J. H. (2017). Levosimendan in Patients with Left Ventricular Dysfunction Undergoing Cardiac Surgery. *The New England Journal of Medicine*, *376*(21), 2032–2042. https://doi.org/10.1056/NEJMOA1616218

Mehta, R. H., van Diepen, S., Meza, J., Bokesch, P., Leimberger, J. D., Tourt-Uhlig, S., Swartz, M., Parrotta, J., Jankowich, R., Hay, D., Harrison, R. W., Fremes, S., Goodman, S. G., Luber, J., Toller, W., Heringlake, M., Anstrom, K. J., Levy, J. H., Harrington, R. A., & Alexander, J. H. (2016). Levosimendan in patients with left ventricular systolic dysfunction undergoing cardiac surgery on cardiopulmonary bypass: Rationale and study design of the Levosimendan in Patients with Left Ventricular Systolic Dysfunction Undergoing Cardiac Surgery Requiring Cardiopulmonary Bypass (LEVO-CTS) trial. *American Heart Journal*, *182*, 62–71. https://doi.org/10.1016/J.AHJ.2016.09.001

Mi, Y. P., Chau, A. K. T., Chiu, C. S. W., Yung, T. C., Lun, K. S., & Cheung, Y. F. (2005). Evolution of the management approach for pulmonary atresia with intact ventricular septum. *Heart (British Cardiac Society)*, *91*(5), 657–663. https://doi.org/10.1136/HRT.2004.033720

Miceli, A., Fiorani, B., Danesi, T. H., Melina, G., & Sinatra, R. (2009). Prophylactic intra-aortic balloon pump in high-risk patients undergoing coronary artery bypass grafting: a propensity score analysis. *Interactive Cardiovascular and Thoracic Surgery*, *9*(2), 291–294. https://doi.org/10.1510/ICVTS.2008.196105

Michalopoulos, A., Stavridis, G., & Geroulanos, S. (1998). Severe sepsis in cardiac surgical patients. *The European Journal of Surgery = Acta Chirurgica*, *164*(3), 217–222. https://doi.org/10.1080/110241598750004670

Michalopoulos, A., Tzelepis, G., Pavlides, G., Kriaras, J., Dafni, U., & Geroulanos, S. (1996). Determinants of duration of ICU stay after coronary artery bypass graft surgery. *British Journal of Anaesthesia*, *77*(2), 208–212. https://doi.org/10.1093/BJA/77.2.208

Møller, C. H., Perko, M. J., Lund, J. T., Andersen, L. W., Kelbæk, H., Madsen, J. K., Winkel, P., Gluud, C., & Steinbrüchel, D. A. (2010). No major differences in 30-day outcomes in high-risk patients randomized to off-pump versus on-pump coronary bypass surgery: the best bypass surgery trial. *Circulation*, *121*(4), 498–504. https://doi.org/10.1161/CIRCULATIONAHA.109.880443

Møller, C. H., Perko, M. J., Lund, J. T., Andersen, L. W., Kelbæk, H., Madsen, J. K., Winkel, P., Gluud, C., & Steinbrüchel, D. A. (2011). Three-year follow-up in a subset of high-risk patients randomly assigned to off-pump versus on-pump coronary artery bypass surgery: the Best Bypass Surgery trial. *Heart (British Cardiac Society)*, *97*(11), 907–913. https://doi.org/10.1136/HRT.2010.211680

Mourad, F., Cleve, N., Nowak, J., Wendt, D., Sander, A., Demircioglu, E., el Gabry, M., Jakob, H., & Shehada, S. E. (2020). Long-Term Single-Center Outcomes of Patients With Chronic Renal Dialysis Undergoing Cardiac Surgery. *The Annals of Thoracic Surgery*, *109*(5), 1442–1448. https://doi.org/10.1016/J.ATHORACSUR.2019.08.042

Nardi, P., Pisano, C., Bertoldo, F., Vacirca, S. R., Saitto, G., Costantino, A., Bovio, E., Pellegrino, A., & Ruvolo, G. (2018). Warm blood cardioplegia versus cold crystalloid cardioplegia for myocardial protection during coronary artery bypass grafting surgery. *Cell Death Discovery*, *4*(1). https://doi.org/10.1038/S41420-018-0031-Z

Nguyen, L. S., Squara, P., Amour, J., Carbognani, D., Bouabdallah, K., Thierry, S., Apert-Verneuil, C., Moyne, A., & Cholley, B. (2018). Intravenous ivabradine versus placebo in patients with low cardiac output syndrome treated by dobutamine after elective coronary artery bypass surgery: a phase 2 exploratory randomized controlled trial. *Critical Care (London, England)*, *22*(1). https://doi.org/10.1186/S13054-018-2124-8

Nicolini, F., Beghi, C., Barbieri, F., Secchi, P., Agostinelli, A., Fragnito, C., Spaggiari, I., & Gherli, T. (2010). Aortic valve replacement in octogenarians: Analysis of risk factors for early and late mortality. *Journal of Heart Valve Disease*, *19*(5), 615–622.

Nicolini, F., Fragnito, C., Molardi, A., Agostinelli, A., Campodonico, R., Spaggiari, I., Beghi, C., & Gherli, T. (2011). Heart surgery in patients on chronic dialysis: Is there still room for improvement in early and long-term outcome? *Heart and Vessels*, *26*(1), 46–54. https://doi.org/10.1007/s00380-010-0024-1

Niedner, M. F., Foley, J. L., Riffenburgh, R. H., Bichell, D. P., Peterson, B. M., & Rodarte, A. (2010). B-type natriuretic peptide: perioperative patterns in congenital heart disease. *Congenital Heart Disease*, *5*(3), 243–255. https://doi.org/10.1111/J.1747-0803.2010.00396.X

Nielsen, D. V., Torp-Pedersen, C., Skals, R. K., Gerds, T. A., Karaliunaite, Z., & Jakobsen, C. J. (2018). Intraoperative milrinone versus dobutamine in cardiac surgery patients: a retrospective cohort study on mortality. *Critical Care (London, England)*, *22*(1). https://doi.org/10.1186/S13054-018-1969-1

Nordness, M. J., Westrick, A. C., Chen, H., & Clay, M. A. (2019). Identification of Low Cardiac Output Syndrome at the Bedside: A Pediatric Cardiac Intensive Care Unit Survey. *Critical Care Nurse*, *39*(2). https://doi.org/10.4037/CCN2019794

Norkiene, I., Ringaitiene, D., Misiuriene, I., Samalavicius, R., Bubulis, R., Baublys, A., & Uzdavinys, G. (2007). Incidence and precipitating factors of delirium after coronary artery bypass grafting. *Scandinavian Cardiovascular Journal : SCJ*, *41*(3), 180–185. https://doi.org/10.1080/14017430701302490

Ok, Y. J., Lim, J. Y., & Jung, S. H. (2018). Critical Illness-Related Corticosteroid Insufficiency in Patients with Low Cardiac Output Syndrome after Cardiac Surgery. *The Korean Journal of Thoracic and Cardiovascular Surgery*, *51*(2), 109–113. https://doi.org/10.5090/KJTCS.2018.51.2.109

Okamura, T., Sunamori, M., Amano, J., Hirooka, Y., Ozeki, M., Tanaka, A., & Suzuki, A. (1984). Combined treatment of coenzyme Q10 and aprotinin with intraaortic balloon pumping following aorto-coronary bypass surgery. *The Japanese Journal of Surgery*, *14*(2), 97–103. https://doi.org/10.1007/BF02469798

Olsson, M., Granström, L., Lindblom, D., Rosenqvist, M., & Rydén, L. (1992). Aortic valve replacement in octogenarians with aortic stenosis: a case-control study. *Journal of the American College of Cardiology*, *20*(7), 1512–1516. https://doi.org/10.1016/0735-1097(92)90444-R

Onem, G., Sacar, M., Baltalarli, A., Ozcan, A. V., Gurses, E., & Sungurtekin, H. (2006). Comparison of simultaneous antegrade/vein graft cardioplegia with antegrade cardioplegia for myocardial protection. *Advances in Therapy*, *23*(6), 869–877. https://doi.org/10.1007/BF02850208

Onorati, F., Biancari, F., de Feo, M., Mariscalco, G., Messina, A., Santarpino, G., Santini, F., Beghi, C., Nappi, G., Troise, G., Fischlein, T., Passerone, G., Heikkinen, J., & Faggian, G. (2015). Mid-term results of aortic valve surgery in redo scenarios in the current practice: results from the multicentre European RECORD (REdo Cardiac Operation Research Database) initiative†. *European Journal of Cardio-Thoracic Surgery : Official Journal of the European Association for Cardio-Thoracic Surgery*, *47*(2), 269–280. https://doi.org/10.1093/EJCTS/EZU116

Osawa, E. A., Rhodes, A., Landoni, G., Galas, F. R. B. G., Fukushima, J. T., Park, C. H. L., Almeida, J. P., Nakamura, R. E., Strabelli, T. M. V., Pileggi, B., Leme, A. C., Fominskiy, E., Sakr, Y., Lima, M., Franco, R. A., Chan, R. P. C., Piccioni, M. A., Mendes, P., Menezes, S. R., … Hajjar, L. A. (2016). Effect of Perioperative Goal-Directed Hemodynamic Resuscitation Therapy on Outcomes Following Cardiac Surgery: A Randomized Clinical Trial and Systematic Review. *Critical Care Medicine*, *44*(4), 724–733. https://doi.org/10.1097/CCM.0000000000001479

Oshima, K., Kunimoto, F., Takahashi, T., Mohara, J., Takeyoshi, I., Hinohara, H., Hayashi, Y., Tajima, Y., & Kuwano, H. (2007). Factors for successful weaning from a percutaneous cardiopulmonary support system (PCPS) in patients with low cardiac output syndrome after cardiovascular surgery. *International Heart Journal*, *48*(6), 743–754. https://doi.org/10.1536/IHJ.48.743

Oualha, M., Urien, S., Spreux-Varoquaux, O., Bordessoule, A., D’Agostino, I., Pouard, P., & Tréluyer, J. M. (2014). Pharmacokinetics, hemodynamic and metabolic effects of epinephrine to prevent post-operative low cardiac output syndrome in children. *Critical Care (London, England)*, *18*(1). https://doi.org/10.1186/CC13707

Pagowska-Klimek, I., Pychynska-Pokorska, M., Krajewski, W., & Moll, J. J. (2011). Predictors of long intensive care unit stay following cardiac surgery in children. *European Journal of Cardio-Thoracic Surgery : Official Journal of the European Association for Cardio-Thoracic Surgery*, *40*(1), 179–184. https://doi.org/10.1016/J.EJCTS.2010.11.038

Pągowska-Klimek, I., Świerzko, A. S., Michalski, M., Głowacka, E., Szala-Poździej, A., Sokołowska, A., Moll, M., Krajewski, W. R., Romak, J., & Cedzyński, M. (2016). Activation of the lectin pathway of complement by cardiopulmonary bypass contributes to the development of systemic inflammatory response syndrome after paediatric cardiac surgery. *Clinical and Experimental Immunology*, *184*(2), 257–263. https://doi.org/10.1111/CEI.12763

Pągowska-Klimek, I., Świerzko, A. S., Michalski, M., Moll, M., Szala-Poździej, A., Sokołowska, A., Krajewski, W. R., & Cedzyński, M. (2016). Mannose-binding lectin (MBL) insufficiency protects against the development of systemic inflammatory response after pediatric cardiac surgery. *Immunobiology*, *221*(2), 175–181. https://doi.org/10.1016/J.IMBIO.2015.09.010

Paparella, D., Scrascia, G., Paramythiotis, A., Guida, P., Magari, V., Malvindi, P. G., Favale, S., & de Luca Tupputi Schinosa, L. (2010). Preoperative cardiac troponin I to assess midterm risks of coronary bypass grafting operations in patients with recent myocardial infarction. *The Annals of Thoracic Surgery*, *89*(3), 696–702. https://doi.org/10.1016/J.ATHORACSUR.2009.11.072

Parmar, D., Lakhia, K., Garg, P., Patel, K., Shah, R., Surti, J., Panchal, J., & Pandya, H. (2017). Risk Factors for Delayed Extubation after Ventricular Septal Defect Closure: a Prospective Observational Study. *Brazilian Journal of Cardiovascular Surgery*, *32*(4), 276–282. https://doi.org/10.21470/1678-9741-2017-0031

Pechlivanidis, K., Onorati, F., Petrilli, G., Santini, F., Milano, A., Torre, S., Calzaferri, D., Mazzucco, A., & Faggian, G. (2014). In which patients is transcatheter aortic valve replacement potentially better indicated than surgery for redo aortic valve disease? Long-term results of a 10-year surgical experience. *The Journal of Thoracic and Cardiovascular Surgery*, *148*(2). https://doi.org/10.1016/J.JTCVS.2013.09.031

Perek, B., Jemielity, M., & Dyszkiewicz, W. (2003). Why are the results of coronary artery bypass grafting in women worse? *Asian Cardiovascular & Thoracic Annals*, *11*(4), 293–298. https://doi.org/10.1177/021849230301100405

Pérez Vela, J. L., Jiménez Rivera, J. J., Alcalá Llorente, M., González de Marcos, B., Torrado, H., García Laborda, C., Fernández Zamora, M. D., González Fernández, F. J., & Martín Benítez, J. C. (2018). Low cardiac output syndrome in the postoperative period of cardiac surgery. Profile, differences in clinical course and prognosis. The ESBAGA study. *Medicina Intensiva*, *42*(3), 159–167. https://doi.org/10.1016/J.MEDIN.2017.05.009

Pérez-Navero, J. L., de la Torre-Aguilar, M. J., Ibarra de la Rosa, I., Gil-Campos, M., Gómez-Guzmán, E., Merino-Cejas, C., Muñoz-Villanueva, M. C., & Llorente-Cantarero, F. J. (2017). Cardiac Biomarkers of Low Cardiac Output Syndrome in the Postoperative Period After Congenital Heart Disease Surgery in Children. *Revista Espanola de Cardiologia (English Ed.)*, *70*(4), 267–274. https://doi.org/10.1016/J.REC.2016.09.011

Pieri, M., Belletti, A., Monaco, F., Pisano, A., Musu, M., Dalessandro, V., Monti, G., Finco, G., Zangrillo, A., & Landoni, G. (2016). Outcome of cardiac surgery in patients with low preoperative ejection fraction. *BMC Anesthesiology*, *16*(1). https://doi.org/10.1186/S12871-016-0271-5

Plicner, D., Stoliński, J., Wąsowicz, M., Gawęda, B., Hymczak, H., Kapelak, B., Drwiła, R., & Undas, A. (2016). Preoperative values of inflammatory markers predict clinical outcomes in patients after CABG, regardless of the use of cardiopulmonary bypass. *Indian Heart Journal*, *68 Suppl 3*(Suppl 3), S10–S15. https://doi.org/10.1016/J.IHJ.2016.10.002

Prifti, E., Bonacchi, M., Frati, G., Giunti, G., Proietti, P., Leacche, M., Massetti, M., Babatasi, G., & Sani, G. (2001). Beating heart myocardial revascularization on extracorporeal circulation in patients with end-stage coronary artery disease. *Cardiovascular Surgery (London, England)*, *9*(6), 608–614. https://doi.org/10.1016/S0967-2109(01)00092-8

Prifti, E., Bonacchi, M., Giunti, G., Frati, G., Proietti, P., Leacche, M., Salica, A., Sani, G., & Brancaccio, G. (2000). Does on-pump/beating-heart coronary artery bypass grafting offer better outcome in end-stage coronary artery disease patients? *Journal of Cardiac Surgery*, *15*(6), 403–410. https://doi.org/10.1111/J.1540-8191.2000.TB01300.X

Probst, S., Cech, C., Haentschel, D., Scholz, M., & Ender, J. (2014). A specialized post anaesthetic care unit improves fast-track management in cardiac surgery: a prospective randomized trial. *Critical Care (London, England)*, *18*(4). https://doi.org/10.1186/S13054-014-0468-2

Radman, M., Keller, R. L., Oishi, P., Datar, S. A., Wellnitz, K., Azakie, A., Hanley, F., Char, D., Hsu, J. H., Amrinovin, R., Adatia, I., & Fineman, J. R. (2014). Preoperative B-type natriuretic peptide levels are associated with outcome after total cavopulmonary connection (Fontan). *The Journal of Thoracic and Cardiovascular Surgery*, *148*(1), 212–219. https://doi.org/10.1016/J.JTCVS.2013.08.009

Raja, S. G., Haider, Z., & Ahmad, M. (2003). Predictors of gastrointestinal complications after conventional and beating heart coronary surgery. *The Surgeon : Journal of the Royal Colleges of Surgeons of Edinburgh and Ireland*, *1*(4), 221–228. https://doi.org/10.1016/S1479-666X(03)80021-5

Ranucci, M., Bozzetti, G., Ditta, A., Cotza, M., Carboni, G., & Ballotta, A. (2008). Surgical reexploration after cardiac operations: why a worse outcome? *The Annals of Thoracic Surgery*, *86*(5), 1557–1562. https://doi.org/10.1016/J.ATHORACSUR.2008.07.114

Ranucci, M., Frigiola, A., Menicanti, L., Ditta, A., Boncilli, A., & Brozzi, S. (2005). Postoperative antithrombin levels and outcome in cardiac operations. *Critical Care Medicine*, *33*(2), 355–360. https://doi.org/10.1097/01.CCM.0000153409.55645.58

Rao, V., Ivanov, J., Weisel, R. D., Cohen, G., Borger, M. A., & Mickle, D. A. G. (2001). Lactate release during reperfusion predicts low cardiac output syndrome after coronary bypass surgery. *The Annals of Thoracic Surgery*, *71*(6), 1925–1930. https://doi.org/10.1016/S0003-4975(01)02634-0

Rao, V., Ivanov, J., Weisel, R. D., Ikonomidis, J. S., Christakis, G. T., & David, T. E. (1996). Predictors of low cardiac output syndrome after coronary artery bypass. *The Journal of Thoracic and Cardiovascular Surgery*, *112*(1), 38–51. https://doi.org/10.1016/S0022-5223(96)70176-9

Remadi, J. P., Rakotoarivelo, Z., Marticho, P., & Benamar, A. (2006). Prospective randomized study comparing coronary artery bypass grafting with the new mini-extracorporeal circulation Jostra System or with a standard cardiopulmonary bypass. *American Heart Journal*, *151*(1), 198.e1-198.e7. https://doi.org/10.1016/J.AHJ.2005.03.067

Renner, A., Zittermann, A., Aboud, A., Hakim-Meibodi, K., Börgermann, J., & Gummert, J. F. (2015). Early and mid-term clinical outcome in younger and elderly patients undergoing mitral valve repair with or without tricuspid valve repair. *Interactive Cardiovascular and Thoracic Surgery*, *20*(1), 85–89. https://doi.org/10.1093/ICVTS/IVU337

Rhodes, L., Erwin, W., … S. B.-P. critical care, & 2017, undefined. (n.d.). Central venous to arterial carbon dioxide difference monitoring after cardiac surgery in infants and neonates. *Ncbi.Nlm.Nih.Gov*. Retrieved June 23, 2022, from https://www.ncbi.nlm.nih.gov/pmc/articles/PMC5336489/

Ricci, Z., Garisto, C., Favia, I., Vitale, V., di Chiara, L., & Cogo, P. E. (2012). Levosimendan infusion in newborns after corrective surgery for congenital heart disease: randomized controlled trial. *Intensive Care Medicine*, *38*(7), 1198–1204. https://doi.org/10.1007/S00134-012-2564-6

Rizza, A., Bignami, E., Belletti, A., Polito, A., Ricci, Z., Isgrò, G., Locatelli, A., & Cogo, P. (2016). Vasoactive Drugs and Hemodynamic Monitoring in Pediatric Cardiac Intensive Care: An Italian Survey. *World Journal for Pediatric & Congenital Heart Surgery*, *7*(1), 25–31. https://doi.org/10.1177/2150135115606626

Robert, S. M., Borasino, S., Dabal, R. J., Cleveland, D. C., Hock, K. M., & Alten, J. A. (2015). Postoperative Hydrocortisone Infusion Reduces the Prevalence of Low Cardiac Output Syndrome After Neonatal Cardiopulmonary Bypass. *Pediatric Critical Care Medicine : A Journal of the Society of Critical Care Medicine and the World Federation of Pediatric Intensive and Critical Care Societies*, *16*(7), 629–636. https://doi.org/10.1097/PCC.0000000000000426

Roberts, A. J., Spies, S. M., Lichtenthal, P. R., Moran, J. M., Sanders, J. H., & Michaelis, L. L. (1983). Changes in left ventricular performance related to perioperative myocardial infarction in coronary artery bypass graft surgery. *The Annals of Thoracic Surgery*, *35*(5), 516–524. https://doi.org/10.1016/S0003-4975(10)60425-0

Rosseel, P. M. J., Santman, F. W., Bouter, H., & Dott, C. S. (1997). Postcardiac surgery low cardiac output syndrome: dopexamine or dopamine? *Intensive Care Medicine*, *23*(9), 962–968. https://doi.org/10.1007/S001340050439

Rosu, C., Laflamme, M., Perrault-Hébert, C., Carrier, M., & Perrault, L. P. (2012). Decreased incidence of low output syndrome with a switch from tepid to cold continuous minimally diluted blood cardioplegia in isolated coronary artery bypass grafting. *Interactive Cardiovascular and Thoracic Surgery*, *15*(4), 655–660. https://doi.org/10.1093/ICVTS/IVS294

Ruokonen, E., Takala, J., & Kari, A. (1993). Regional blood flow and oxygen transport in patients with the low cardiac output syndrome after cardiac surgery. *Critical Care Medicine*, *21*(9), 1304–1311. https://doi.org/10.1097/00003246-199309000-00012

Rzucidło‑Resil, J., Plicner, D., Gackowski, A., Kapelak, B., & Stoliński, J. (2019). Impact of the mechanism of mitral regurgitation on clinical outcomes in patients after mitral valve surgery. *Kardiologia Polska*, *77*(5), 525–534. https://doi.org/10.5603/KP.A2019.0043

Sá, M. P. B. D. O., Soares, E. F., Santos, C. A., Figueiredo, O. J., Lima, R. O. A., Escobar, R. R., de Rueda, F. G., & Lima, R. de C. (2012). Perioperative mortality in diabetic patients undergoing coronary artery bypass graft surgery. *Revista Do Colegio Brasileiro de Cirurgioes*, *39*(1), 22–27. https://doi.org/10.1590/S0100-69912012000100006

Sá, M. P. B. de O., Nogueira, J. R. C., Ferraz, P. E., Figueiredo, O. J., Cavalcante, W. C. P., Cavalcante, T. C. P., da Silva, H. T. T., Santos, C. A., Lima, R. O. de A., Vasconcelos, F. P., & Lima, R. de C. (2012). Risk factors for low cardiac output syndrome after coronary artery bypass grafting surgery. *Revista Brasileira de Cirurgia Cardiovascular : Orgao Oficial Da Sociedade Brasileira de Cirurgia Cardiovascular*, *27*(2), 217–223. https://doi.org/10.5935/1678-9741.20120037

Sabzi, F., & Faraji, R. (2015). Predictors of post pericardiotomy low cardiac output syndrome in patients with pericardial effusion. *Journal of Cardiovascular and Thoracic Research*, *7*(1), 18–23. https://doi.org/10.15171/JCVTR.2015.04

Sano, S., Nawa, S., Senoo, Y., & Teramoto, S. (1987). Echocardiographic prediction of postoperative low cardiac output syndrome in patients with mitral stenosis. *Acta Medica Okayama*, *41*(5), 215–222. https://doi.org/10.18926/AMO/31757

Sato, N., Uchida, N., Miura, M., Ohmi, M., Fukuju, T., Tabayashi, K., Haneda, K., & Mohri, H. (1993). Risk analysis of low cardiac output syndrome after valve replacement. *The Tohoku Journal of Experimental Medicine*, *171*(1), 77–88. https://doi.org/10.1620/TJEM.171.77

Savas Oz, B., Arslan, G., Kaya, E., Gunay, C., Cingoz, F., & Arslan, M. (2013). Comparison of results of autologous versus homologous blood transfusion in open-heart surgery. *Cardiovascular Journal of Africa*, *24*(4). https://doi.org/10.5830/CVJA-2013-020

Saylam, A., Melo, J. Q., Ahmad, A., Chapman, R. D., Wood, J. A., & Starr, A. (1978). Pulmonary embolectomy. *The Western Journal of Medicine*, *128*(5), 377–381. https://doi.org/10.1177/153857446900300103

Schlapbach, L. J., Horton, S. B., Long, D. A., Beca, J., Erickson, S., Festa, M., D’Udekem, Y., Alphonso, N., Winlaw, D., Johnson, K., Delzoppo, C., van Loon, K., Gannon, B., Fooken, J., Blumenthal, A., Young, P., Jones, M., Butt, W., & Schibler, A. (2019). Study protocol: NITric oxide during cardiopulmonary bypass to improve Recovery in Infants with Congenital heart defects (NITRIC trial): a randomised controlled trial. *BMJ Open*, *9*(8). https://doi.org/10.1136/BMJOPEN-2018-026664

Shah, B., Sharma, P., Brahmbhatt, A., Shah, R., Rathod, B., Shastri, N., Patel, J., & Malhotra, A. (2014). Study of levosimendan during off-pump coronary artery bypass grafting in patients with LV dysfunction: a double-blind randomized study. *Indian Journal of Pharmacology*, *46*(1), 29–34. https://doi.org/10.4103/0253-7613.125161

Sharma, P., Malhotra, A., Gandhi, S., Garg, P., Bishnoi, A., & Gandhi, H. (2014). Preoperative levosimendan in ischemic mitral valve repair. *Asian Cardiovascular & Thoracic Annals*, *22*(5), 539–545. https://doi.org/10.1177/0218492313499352

Shi, S., Zhao, Z., Liu, X., Shu, Q., Tan, L., Lin, R., Shi, Z., & Fang, X. (2008). Perioperative risk factors for prolonged mechanical ventilation following cardiac surgery in neonates and young infants. *Chest*, *134*(4), 768–774. https://doi.org/10.1378/CHEST.07-2573

Shida, H., Morimoto, M., Inokawa, K., & Kuroda, T. (1981). Changes of kallikrein and serotonin in plasma during and after open-heart surgery. *Japanese Circulation Journal*, *45*(1), 48–54. https://doi.org/10.1253/JCJ.45.48

Shingu, Y., Ooka, T., Katoh, H., Tachibana, T., Kubota, S., & Matsui, Y. (2018). Feasibility and limitations of mitral valve repair, with or without left ventricular reconstruction in non-ischemic dilated cardiomyopathy. *Journal of Cardiology*, *71*(4), 329–335. https://doi.org/10.1016/J.JJCC.2017.09.013

Sinatra, R., MacRina, F., Braccio, M., Melina, G., Luzi, G., Ruvolo, G., & Marino, B. (1997). Left ventricular aneurysmectomy; comparison between two techniques; early and late results. *European Journal of Cardio-Thoracic Surgery : Official Journal of the European Association for Cardio-Thoracic Surgery*, *12*(2), 291–297. https://doi.org/10.1016/S1010-7940(97)00121-8

Sobieraj, M., Kilanowska, M., Ladzinski, P., Garbuzowa, I., Wojtalik, M., Moczko, J., & Mrówczynski, W. (2018). Type of cardioplegic solution as a factor influencing the clinical outcome of open-heart congenital procedures. *Kardiochirurgia i Torakochirurgia Polska = Polish Journal of Cardio-Thoracic Surgery*, *15*(2), 86–94. https://doi.org/10.5114/KITP.2018.76473

Spiliotopoulos, K., Maganti, M., Brister, S., & Rao, V. (2011). Changing pattern of reoperative coronary artery bypass grafting: a 20-year study. *The Annals of Thoracic Surgery*, *92*(1), 40–47. https://doi.org/10.1016/J.ATHORACSUR.2011.03.104

Stamou, S. C., Hill, P. C., Dangas, G., Pfister, A. J., Boyce, S. W., Dullum, M. K. C., Bafi, A. S., & Corso, P. J. (2001). Stroke after coronary artery bypass: incidence, predictors, and clinical outcome. *Stroke*, *32*(7), 1508–1512. https://doi.org/10.1161/01.STR.32.7.1508

Stromberg, D., Raymond, T., Samuel, D., Crockford, D., Stigall, W., Leonard, S., Mendeloff, E., & Gormley, A. (2012). Use of the cardioprotectants thymosin β4 and dexrazoxane during congenital heart surgery: proposal for a randomized, double-blind, clinical trial. *Annals of the New York Academy of Sciences*, *1270*(1), 59–65. https://doi.org/10.1111/J.1749-6632.2012.06710.X

Sunny, Yunus, M., Karim, H. M. R., Saikia, M. K., Bhattacharyya, P., & Dey, S. (2016). Comparison of Levosimendan, Milrinone and Dobutamine in treating Low Cardiac Output Syndrome Following Valve Replacement Surgeries with Cardiopulmonary Bypass. *Journal of Clinical and Diagnostic Research : JCDR*, *10*(12), UC05–UC08. https://doi.org/10.7860/JCDR/2016/23584.8987

Suominen, P. K., Dickerson, H. A., Moffett, B. S., Ranta, S. O., Mott, A. R., Price, J. F., Heinle, J. S., McKenzie, E. D., Fraser, C. D., & Chang, A. C. (2005). Hemodynamic effects of rescue protocol hydrocortisone in neonates with low cardiac output syndrome after cardiac surgery. *Pediatric Critical Care Medicine : A Journal of the Society of Critical Care Medicine and the World Federation of Pediatric Intensive and Critical Care Societies*, *6*(6), 655–659. https://doi.org/10.1097/01.PCC.0000185487.69215.29

Thielmann, M., Massoudy, P., Neuhäuser, M., Knipp, S., Kamler, M., Marggraf, G., Piotrowski, J., & Jakob, H. (2005). Risk stratification with cardiac troponin I in patients undergoing elective coronary artery bypass surgery. *European Journal of Cardio-Thoracic Surgery : Official Journal of the European Association for Cardio-Thoracic Surgery*, *27*(5), 861–869. https://doi.org/10.1016/J.EJCTS.2005.01.043

Thielmann, M., Massoudy, P., Neuhäuser, M., Knipp, S., Kamler, M., Piotrowski, J., Mann, K., & Jakob, H. (2005). Prognostic value of preoperative cardiac troponin I in patients with non-ST-segment elevation acute coronary syndromes undergoing coronary artery bypass surgery. *Chest*, *128*(5), 3526–3536. https://doi.org/10.1378/CHEST.128.5.3526

Thielmann, M., Massoudy, P., Neuhäuser, M., Tsagakis, K., Marggraf, G., Kamler, M., Mann, K., Erbel, R., & Jakob, H. (2006). Prognostic value of preoperative cardiac troponin I in patients undergoing emergency coronary artery bypass surgery with non-ST-elevation or ST-elevation acute coronary syndromes. *Circulation*, *114*(1 Suppl). https://doi.org/10.1161/CIRCULATIONAHA.105.001057

Tolpin, D. A., Collard, C. D., Lee, V. V., Elayda, M. A., & Pan, W. (2009). Obesity is associated with increased morbidity after coronary artery bypass graft surgery in patients with renal insufficiency. *The Journal of Thoracic and Cardiovascular Surgery*, *138*(4), 873–879. https://doi.org/10.1016/J.JTCVS.2009.02.019

Tóth, R., Breuer, T., Cserép, Z., Lex, D., Fazekas, L., Sápi, E., Szatmári, A., Gál, J., & Székely, A. (2012). Acute kidney injury is associated with higher morbidity and resource utilization in pediatric patients undergoing heart surgery. *The Annals of Thoracic Surgery*, *93*(6), 1984–1990. https://doi.org/10.1016/J.ATHORACSUR.2011.10.046

Treskatsch, S., Balzer, F., Geyer, T., Spies, C. D., Kastrup, M., Grubitzsch, H., Wernecke, K. D., Erb, J. M., Braun, J. P., & Sander, M. (2015). Early levosimendan administration is associated with decreased mortality after cardiac surgery. *Journal of Critical Care*, *30*(4), 859.e1-859.e6. https://doi.org/10.1016/J.JCRC.2015.03.008

Tritapepe, L., Voci, P., Cogliati, A. A., Pasotti, E., Papalia, U., & Menichetti, A. (1999). Successful weaning from cardiopulmonary bypass with central venous prostaglandin E1 and left atrial norepinephrine infusion in patients with acute pulmonary hypertension. *Critical Care Medicine*, *27*(10), 2180–2183. https://doi.org/10.1097/00003246-199910000-00018

Tsai, Y. T., Lin, F. Y., Lai, C. H., Lin, Y. C., Lin, C. Y., & Tsai, C. S. (2012). On-pump beating-heart coronary artery bypass provides efficacious short- and long-term outcomes in hemodialysis patients. *Nephrology, Dialysis, Transplantation : Official Publication of the European Dialysis and Transplant Association - European Renal Association*, *27*(5), 2059–2065. https://doi.org/10.1093/NDT/GFR536

Udayasankar, S. (2017). Question 2 Is levosimendan better than milrinone in preventing post operative low cardiac output syndrome and improving cardiac function in children with congenital heart disease? *Archives of Disease in Childhood*, *102*(6), 590–592. https://doi.org/10.1136/ARCHDISCHILD-2017-312660

Ulate, K. P., Yanay, O., Jeffries, H., Baden, H., di Gennaro, J. L., & Zimmerman, J. (2017). An Elevated Low Cardiac Output Syndrome Score Is Associated With Morbidity in Infants After Congenital Heart Surgery. *Pediatric Critical Care Medicine : A Journal of the Society of Critical Care Medicine and the World Federation of Pediatric Intensive and Critical Care Societies*, *18*(1), 26–33. https://doi.org/10.1097/PCC.0000000000000979

Umakanthan, R., Leacche, M., Petracek, M. R., Kumar, S., Solenkova, N. v., Kaiser, C. A., Greelish, J. P., Balaguer, J. M., Ahmad, R. M., Ball, S. K., Hoff, S. J., Absi, T. S., Kim, B. S., & Byrne, J. G. (2008). Safety of minimally invasive mitral valve surgery without aortic cross-clamp. *The Annals of Thoracic Surgery*, *85*(5), 1544–1550. https://doi.org/10.1016/J.ATHORACSUR.2008.01.099

Umminger, J., Reitz, M., Rojas, S. v., Stiefel, P., Shrestha, M., Haverich, A., Ismail, I., & Martens, A. (2016). Does the surgeon’s experience have an impact on outcome after total arterial revascularization with composite T-grafts? A risk factor analysis. *Interactive Cardiovascular and Thoracic Surgery*, *23*(5), 749–756. https://doi.org/10.1093/ICVTS/IVW207

van Diepen, S., Mehta, R. H., Leimberger, J. D., Goodman, S. G., Fremes, S., Jankowich, R., Heringlake, M., Anstrom, K. J., Levy, J. H., Luber, J., Nagpal, A. D., Duncan, A. E., Argenziano, M., Toller, W., Teoh, K., Knight, J. D., Lopes, R. D., Cowper, P. A., Mark, D. B., & Alexander, J. H. (2020). Levosimendan in patients with reduced left ventricular function undergoing isolated coronary or valve surgery. *The Journal of Thoracic and Cardiovascular Surgery*, *159*(6), 2302-2309.e6. https://doi.org/10.1016/J.JTCVS.2019.06.020

Verweij, E. J., Hogenbirk, K., Roest, A. A. W., van Brempt, R., Hazekamp, M. G., & de Jonge, E. (2012). Serum cortisol concentration with exploratory cut-off values do not predict the effects of hydrocortisone administration in children with low cardiac output after cardiac surgery. *Interactive Cardiovascular and Thoracic Surgery*, *15*(4), 685–689. https://doi.org/10.1093/ICVTS/IVS292

Vogt, W. (2014). Evaluation and optimisation of current milrinone prescribing for the treatment and prevention of low cardiac output syndrome in paediatric patients after open heart surgery using a physiology-based pharmacokinetic drug-disease model. *Clinical Pharmacokinetics*, *53*(1), 51–72. https://doi.org/10.1007/S40262-013-0096-Z

Wang, A., Cui, C., Fan, Y., Zi, J., Zhang, J., Wang, G., Wang, F., Wang, J., & Tan, Q. (2019). Prophylactic use of levosimendan in pediatric patients undergoing cardiac surgery: a prospective randomized controlled trial. *Critical Care (London, England)*, *23*(1). https://doi.org/10.1186/S13054-019-2704-2

Wang, J., Wang, C., Wang, Y., Gao, Y., Tian, Y., Wang, S., Li, J., Yang, L., Peng, Y. G., & Yan, F. (2020). Fluid Overload in Special Pediatric Cohorts With Anomalous Origin of the Left Coronary Artery From the Pulmonary Artery Following Surgical Repair. *Journal of Cardiothoracic and Vascular Anesthesia*, *34*(6), 1565–1572. https://doi.org/10.1053/J.JVCA.2019.10.013

Wang, L., Hao, X., Wang, X., Gu, C., Wang, H., & Hou, X. (2018a). Short-term outcomes of preoperative intra-aortic balloon pump use in patients undergoing adjunctive coronary endarterectomy: a retrospective observational study. *Perfusion*, *33*(6), 426–432. https://doi.org/10.1177/0267659118759594

Wang, L., Hao, X., Wang, X., Gu, C., Wang, H., & Hou, X. (2018b). Short-term outcomes of preoperative intra-aortic balloon pump use in patients undergoing adjunctive coronary endarterectomy: a retrospective observational study. *Perfusion*, *33*(6), 426–432. https://doi.org/10.1177/0267659118759594

Watkins, K. J., Geisler, S., Doman, T., Orringer, M. J., Tracy, P., Yu, S., Wilder, N. S., & Russell, M. W. (2020). Rise in Angiopoietin-2 Following Neonatal Cardiac Surgery Is Associated With Adverse Clinical Outcomes. *Pediatric Critical Care Medicine : A Journal of the Society of Critical Care Medicine and the World Federation of Pediatric Intensive and Critical Care Societies*, *21*(9), E827–E833. https://doi.org/10.1097/PCC.0000000000002479

WHITE, D. A., LATIMER, R. D., & ODURO, A. (1990). Management of low cardiac output syndrome after cardiac surgery using enoximone. *Anaesthesia*, *45*(5), 386–389. https://doi.org/10.1111/J.1365-2044.1990.TB14783.X

Xu, J., Jiang, W., Li, Y., Shen, B., Shen, Z., Wang, Y., Hu, J., Fang, Y., Luo, Z., Wang, C., Teng, J., Ding, X., & Yu, J. (2020). Volume-associated hemodynamic variables for prediction of cardiac surgery-associated acute kidney injury. *Clinical and Experimental Nephrology*, *24*(9), 798–805. https://doi.org/10.1007/S10157-020-01908-6

Yildirim, A., Güzelmeriç, F., Öner, C. N., Karaaǧaç, A. T., Şaşmazel, A., Erdem, H., Özdemir, O., & Baysal, A. (2014). Prognostic significance of sICAM-1 and sVCAM-1 molecules for cardiac surgery in pediatric patients with pulmonary hypertension. *Anadolu Kardiyoloji Dergisi : AKD = the Anatolian Journal of Cardiology*, *14*(3), 274–279. https://doi.org/10.5152/AKD.2013.4543

Yıldırım, A., Güzelmeriç, F., Oner, C. N., Türkmen Karaağaç, A., Saşmazel, A., Erdem, H., Ozdemir, O., & Baysal, A. (2014). Prognostic significance of sICAM-1 and sVCAM-1 molecules for cardiac surgery in pediatric patients with pulmonary hypertension. *Anadolu Kardiyoloji Dergisi : AKD = the Anatolian Journal of Cardiology*. https://doi.org/10.5152/AKD.2014.4543

Yogaratnam, J. Z., Laden, G., Guvendik, L., Cowen, M., Cale, A., & Griffin, S. (2010). Hyperbaric oxygen preconditioning improves myocardial function, reduces length of intensive care stay, and limits complications post coronary artery bypass graft surgery. *Cardiovascular Revascularization Medicine : Including Molecular Interventions*, *11*(1), 8–19. https://doi.org/10.1016/J.CARREV.2009.03.004

Yoo, J. S., Kim, J. B., Joo, Y., Lee, W. Y., Jung, S. H., Choo, S. J., Chung, C. H., & Lee, J. W. (2014). Deep hypothermic circulatory arrest versus non-deep hypothermic beating heart strategy in descending thoracic or thoracoabdominal aortic surgery. *European Journal of Cardio-Thoracic Surgery : Official Journal of the European Association for Cardio-Thoracic Surgery*, *46*(4), 678–684. https://doi.org/10.1093/EJCTS/EZU053

Yoo, J. S., Kim, J. B., Jung, S. H., Choo, S. J., Chung, C. H., & Lee, J. W. (2014). Surgical repair of descending thoracic and thoracoabdominal aortic aneurysm involving the distal arch: open proximal anastomosis under deep hypothermia versus arch clamping technique. *The Journal of Thoracic and Cardiovascular Surgery*, *148*(5), 2101–2107. https://doi.org/10.1016/J.JTCVS.2014.06.068

Yuan, X., Li, B., Yang, Y., Wang, H., Sun, H., Song, Y., & Wang, W. (2020). Surgical results and pathological analysis of cardiac fibroma in the adolescent and the adult. *Journal of Cardiac Surgery*, *35*(8), 1912–1919. https://doi.org/10.1111/JOCS.14790

Zangrillo, A., Alvaro, G., Pisano, A., Guarracino, F., Lobreglio, R., Bradic, N., Lembo, R., Gianni, S., Calabrò, M. G., Likhvantsev, V., Grigoryev, E., Buscaglia, G., Pala, G., Auci, E., Amantea, B., Monaco, F., de Vuono, G., Corcione, A., Galdieri, N., … Landoni, G. (2016). A randomized controlled trial of levosimendan to reduce mortality in high-risk cardiac surgery patients (CHEETAH): Rationale and design. *American Heart Journal*, *177*, 66–73. https://doi.org/10.1016/J.AHJ.2016.03.021

Zangrillo, A., Maj, G., Monaco, F., Scandroglio, A. M., Nuzzi, M., Plumari, V., Virzo, I., Bignami, E., Casiraghi, G., & Landoni, G. (2010). Cardiac index validation using the pressure recording analytic method in unstable patients. *Journal of Cardiothoracic and Vascular Anesthesia*, *24*(2), 265–269. https://doi.org/10.1053/J.JVCA.2009.09.019

Zarragoikoetxea, I., Vicente, R., Pajares, A., Carmona, P., Lopez, M., Moreno, I., Argente, P., Hornero, F., Valera, F., & Aguero, J. (2020). Quantitative Transthoracic Echocardiography of the Response to Dobutamine in Cardiac Surgery Patients With Low Cardiac Output Syndrome. *Journal of Cardiothoracic and Vascular Anesthesia*, *34*(1), 87–96. https://doi.org/10.1053/J.JVCA.2019.08.019

Zhang, G., Wu, N., Liu, H., Lv, H., Yao, Z., & Li, J. (2009). Case control study of gastrointestinal complications after cardiopulmonary bypass heart surgery. *Perfusion*, *24*(3), 173–178. https://doi.org/10.1177/0267659109346665

Zhang, J., Lang, Y., Guo, L., Song, X., Shu, L., Su, G., Liu, H., & Xu, J. (2015). Preventive use of intra-aortic balloon pump in patients undergoing high-risk coronary artery bypass grafting: a retrospective study. *Medical Science Monitor : International Medical Journal of Experimental and Clinical Research*, *21*, 855–860. https://doi.org/10.12659/MSM.893021

Zhang, L., Jin, Y., Zhang, F., Li, H., & Wu, Q. (2018). Modified Peritoneal Dialysis for Treatment of Acute Renal Failure after Complex Congenital Heart Surgery in Infants. *The Heart Surgery Forum*, *21*(4), E286–E289. https://doi.org/10.1532/HSF.1915

Zhao, K., Zhang, Y., Li, J., Cui, Q., Zhao, R., Chen, W., Liu, J., Zhao, B., Wan, Y., Ma, X. L., Yu, S., Yi, D., & Gao, F. (2020). Modified Glucose-Insulin-Potassium Regimen Provides Cardioprotection With Improved Tissue Perfusion in Patients Undergoing Cardiopulmonary Bypass Surgery. *Journal of the American Heart Association*, *9*(6). https://doi.org/10.1161/JAHA.119.012376

Zhou, W., Wang, G., Liu, Y., Tao, Y., Du, Z., Tang, Y., Qiao, F., Liu, Y., & Xu, Z. (2019). Outcomes and risk factors of postoperative hepatic dysfunction in patients undergoing acute type A aortic dissection surgery. *Journal of Thoracic Disease*, *11*(8), 3225–3233. https://doi.org/10.21037/JTD.2019.08.72
